# Lipid overload-induced RTN3 activation leads to cardiac dysfunction by promoting lipid droplet biogenesis

**DOI:** 10.1038/s41418-023-01241-x

**Published:** 2023-11-28

**Authors:** Dong Guo, Mingming Zhang, Bingchao Qi, Tingwei Peng, Mingchuan Liu, Zhelong Li, Feng Fu, Yanjie Guo, Congye Li, Ying Wang, Lang Hu, Yan Li

**Affiliations:** 1 Department of Cardiology, Tangdu Hospital, Airforce Medical University, Xi’an, 710032 China; 2 Department of Ultrasound Diagnostics, Tangdu Hospital, Airforce Medical University, Xi’an, 710032 China; 3 Department of Physiology and Pathophysiology, Airforce Medical University, Xi’an, 710032 China; 4 Department of Cardiology, Xi’an International Medical Center Hospital, Xi’an, 710100 China; 5 Department of Cardiology, Xijing Hospital, Airforce Medical University, 710032 Xi’an, China

**Keywords:** Glycerides, Cardiomyopathies, Metabolic disorders, Metabolic pathways

## Abstract

Lipid droplet (LD) accumulation is a notable feature of obesity-induced cardiomyopathy, while underlying mechanism remains poorly understood. Here we show that mice fed with high-fat diet (HFD) exhibited significantly increase in cardiac LD and RTN3 expression, accompanied by cardiac function impairment. Multiple loss- and gain-of function experiments indicate that RTN3 is critical to HFD-induced cardiac LD accumulation. Mechanistically, RTN3 directly bonds with fatty acid binding protein 5 (FABP5) to facilitate the directed transport of fatty acids to endoplasmic reticulum, thereby promoting LD biogenesis in a diacylglycerol acyltransferase 2 dependent way. Moreover, lipid overload-induced RTN3 upregulation is due to increased expression of CCAAT/enhancer binding protein α (C/EBPα), which positively regulates RTN3 transcription by binding to its promoter region. Notably, above findings were verified in the myocardium of obese patients. Our findings suggest that manipulating LD biogenesis by modulating RTN3 may be a potential strategy for treating cardiac dysfunction in obese patients.

## Introduction

The global prevalence of obesity has nearly tripled since 1975, and over 1 billion people will become obese by 2025 at the current rate of increase [1]. In 2015, the elevation of body mass index (BMI) contributed to 4.0 million deaths, and cardiovascular disease was the leading cause [2]. Cohort studies have found that obese patients are at a higher risk of developing cardiomyopathy, and severely obese patients (BMI ≥ 35 kg/m^2^) have an approximately fivefold higher risk [3, 4]. Lipids are the main energy source of the adult heart, the oxidation of which fuels 60–80% of the cardiac energy demand [5]. However, lipid accumulation, the hallmark of the obese heart, is a determinant of heart function. Multiple studies have revealed the presence of excess lipids in the hearts of obese individuals, and the extent of lipid accumulation is directly related to the impairment of left ventricular (LV) function [6, 7]. Moreover, genetically modified mice with increased myocardial lipid content developed severe cardiomyopathy and cardiac dysfunction [8, 9]. Although the relationship between myocardial lipid accumulation and cardiac dysfunction has been elucidated, the biological pathways responsible for lipid accumulation in the heart remain largely unknown. Additionally, the mechanism by which obesity promotes myocardial lipid accumulation and leads to cardiac dysfunction remains unclear.

Lipid droplets (LDs) are key organelles responsible for lipid storage and mobilization. Within the normal heart, few LDs are present because of well-matched lipid uptake and oxidation. However, under pathological conditions, especially in the hearts of obese individuals, a dramatic increase in the number and volume of LDs, which are markers of lipid overload, was observed. It has also been reported that aberrant accumulation of LDs is responsible for lipid metabolism disorders and dysregulated metabolic gene transcription, leading to increased susceptibility to heart failure and ischemic heart disease [10]. In general, a pathological increase of LDs in cardiomyocytes may result from increased LD biogenesis, decreased lipid oxidation, or a combination of both. However, specifical to obese individuals, excessive circulating lipid supply leads to dramatically increased cardiac lipid uptake, making overactivated LD biogenesis a major contributor to aberrant lipid accumulation in heart of obese patients [11]. To the best of our knowledge, LD biogenesis occurs primarily in the endoplasmic reticulum (ER) [12]. When lipid uptake is greater than consumption, excessive fatty acids (FAs) are transported to the ER and esterified to synthesize neutral lipids. As the concentration of neutral lipids increases, LDs bud from the ER, grow to become mature, and detach from the ER. The entire process of LD biogenesis in the ER relies on a precisely controlled network of multiple proteins and signaling pathways, such as seipin and the fat storage-inducing transmembrane (FIT) family. Despite large progress has been achieved in the last decades, many molecular details of LD biogenesis, especially the initial stage of this process, are still unclear. Moreover, the regulatory mechanism of LDs biogenesis in the hearts of obese individuals and its role in lipid overload-induced cardiac dysfunction remain unrevealed.

Reticulon 3 (RTN3), a member of RTNs family, was first discovered as a neuroendocrine-specific protein, and dysregulation of RTN3 participated in the development of several neurological diseases. Biochemically, the unique transmembrane structure of RTN3 enables it as an ideal candidate to function at ER membrane and thereby maintain ER function. For example, RTN3 interacts with the endosome surface protein Rab9a and promotes endosome maturation in the ER [13]. ER-assisted endocytosis also depends on tethering between the plasma membrane and ER formed by RTN3 [14]. Interestingly, it was recently discovered that RTN3 was involved in pathogenesis of obesity, in which overexpression of RTN3 induced significant accumulation of LD in adipocytes [15]. Besides, recent studies revealed that RTN3 is broadly expressed in various tissues including the heart [16, 17]. However, whether RTN3 is involved in ER-located LD biogenesis and cardiac LD accumulation, has never been revealed. Here, in this study, we showed that upregulated RTN3 is a novel critical factor contributing to pathologically activated LD biogenesis and lipid accumulation in the hearts of high-fat diet (HFD)-fed mice. Mechanistically, RTN3 directly interacts with fatty acid binding protein 5 (FABP5), which facilitates the transport of FA to ER and promotes LD biogenesis in cardiomyocytes. Moreover, we identified CCAAT/enhancer binding protein α (C/EBPα) as a direct transcriptional factor of RTN3 implicated in lipid overload-induced RTN3 upregulation. Our work uncovered a novel mechanism of LD biogenesis and revealed that RTN3-mediated LD biogenesis plays a pivotal role in the pathogenesis of HFD-induced cardiac lipid accumulation and dysfunction.

## Results

### Excessive LD accumulation and increased RTN3 expression were observed in the hearts of HFD-fed mice

**Fig. 1:**
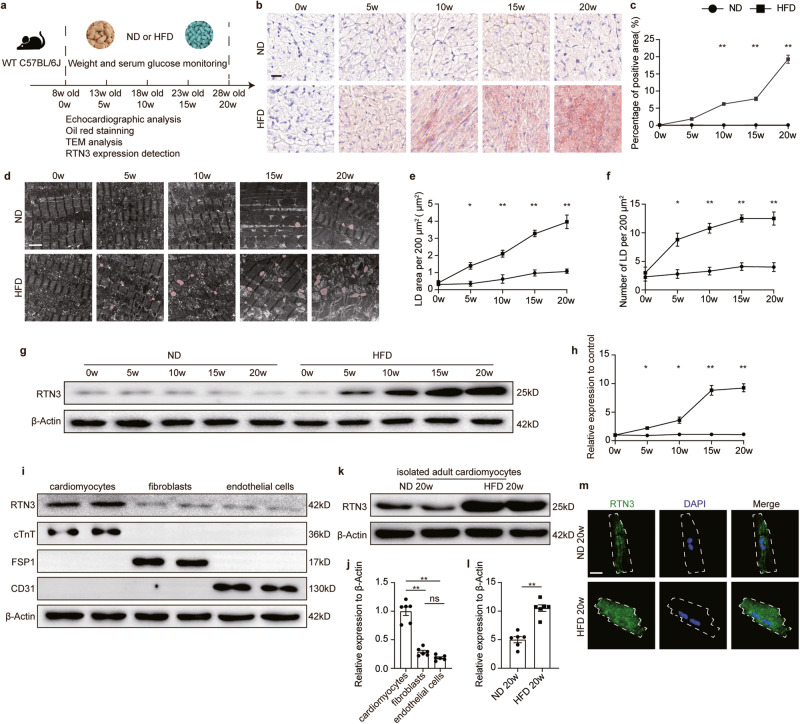
Excessive LD accumulation and increased RTN3 expression were observed in the hearts of HFD-fed mice. **a** Diagram of experimental protocol; **b** Representative oil red O staining images indicating intramyocardial lipid content, scale bar=20μm; **c** Quantitative analysis of positive area of oil red staining (*n* = 10 images each group); **d** Representative TEM images of myocardium, LDs were labeled as red. Scale bar = 2 μm; Quantitative analysis of LD area per 200 μm^2^ (**e**) and LD number per 200 μm^2^ (**f**) (*n* = 10 images each group); Representative western blotting images (**g**) and quantitative analysis (**h**) of RTN3 protein expression in the heart (*n* = 6 mice each group); Representative western blotting images (**i**) and quantitative analysis (**j**) of RTN3 protein expression in cardiomyocytes, cardiac fibroblasts, and endothelial cells (*n* = 6 each group); Representative western blotting images (**k**) and quantitative analysis (**l**) of RTN3 protein expression in isolated adult cardiomyocytes (*n* = 6 mice each group); **m** Representative immunofluorescence images indicating RTN3 and nucleus in isolated adult cardiomyocytes, scale bar = 10 μm. Data are expressed as Mean ± SEM. Differences are significant for *P < 0.05, **P < 0.01.

Wild-type (WT) C57BL/6J mice were fed with HFD continuously (60 kcal% fat) to induce obesity (Fig. 1a). Compared with mice fed with normal diet (ND), HFD-fed mice exhibited a significant increase in body weight, 12 h fasting blood glucose, and serum lipids [including triglycerides (TG), cholesterol (CHO), high-density lipoprotein (HDL), and low-density lipoprotein (LDL)] (Supplementary Fig. 1a–f). Serial echocardiography was used to monitor the effect of HFD on cardiac function (Supplementary Fig. 1g–k and Supplementary Table 1). After 10 weeks of feeding, diastolic LV function in HFD-fed mice showed significant impairment (E/A ratio decreased from 1.8 to 1.5, Supplementary Fig. 1g and h), while systolic function remained unaffected (Supplementary Fig. 1i–k). After feeding for 15 and 20 weeks, both systolic and diastolic LV function in HFD-fed mice exhibited a significant decrease, as indicated by decreased left ventricular ejection fraction (LVEF), left ventricular fractional shortening (LVFS), and E/A ratio (Supplementary Fig. 1g–k). Moreover, after feeding for 20 weeks, heart weight (HW)/ tibia length (TL) and area of isolated adult cardiomyocytes was increased in HFD-fed mice (Supplementary Fig. 1l–m). Cardiac lipid content was detected using Oil Red O staining and transmission electron microscopy (TEM). As shown in Fig. 1b, Oil Red O staining revealed a significant increase in cardiac lipids in the hearts of HFD mice, especially in 20-week HFD-fed mice (Fig. 1b, c). In addition, TEM results demonstrated that myocardial LDs content increased dramatically in HFD-fed mice compared to that in ND-fed mice (Fig. 1d). Specifically, LD area, LD number, and average LD area increased with the extension of HFD time (Fig. 1d–f, Supplementary Fig. 1n). Further analysis of the LD diameter distribution revealed that the proportion of large LDs (diameter >800 nm) was significantly elevated in the hearts of mice after 20 weeks of HFD feeding (Supplementary Fig. 1o). Collectively, these results demonstrated that HFD caused time-dependent cardiac dysfunction accompanied by myocardial lipid accumulation.

To investigate whether RTN3 was involved in HFD-induced cardiac impairment, RTN3 expression was detected in the cardiac tissue of HFD-fed mice at different time points. Western blotting revealed that HFD feeding significantly increased the protein level of RTN3 in the heart (Fig. 1g, h). To further determine which cell type in the heart was responsible for the RTN3 upregulation, RTN3 expression in neonatal cardiomyocytes, cardiac fibroblasts, and endothelial cells were detected respectively. As shown in Fig. 1i, j, cardiomyocytes were the predominant cells in the hearts expressing RTN3, while the two other cell types showed almost negligible RTN3 expression. Furthermore, adult cardiomyocytes were isolated from heart of ND or HFD-fed mice. Western blotting and immunofluorescence results both demonstrated that RTN3 in adult cardiomyocytes was obviously upregulated upon HFD feeding (Fig. 1k–m). Moreover, correlation analysis results revealed that RTN3 levels were positively correlated with myocardial LD content, but negatively correlated with cardiac function indicators, including LVEF, LVFS, and E/A ratio (Supplementary Fig. 1p–s). These data suggest a strong relationship between RTN3 upregulation and pathological cardiac lipid storage as well as cardiac function impairment in the hearts of obese mice.

### Cardiomyocyte-specific RTN3 overexpression led to myocardial lipid accumulation and cardiac dysfunction in ND-fed mice

**Fig. 2:**
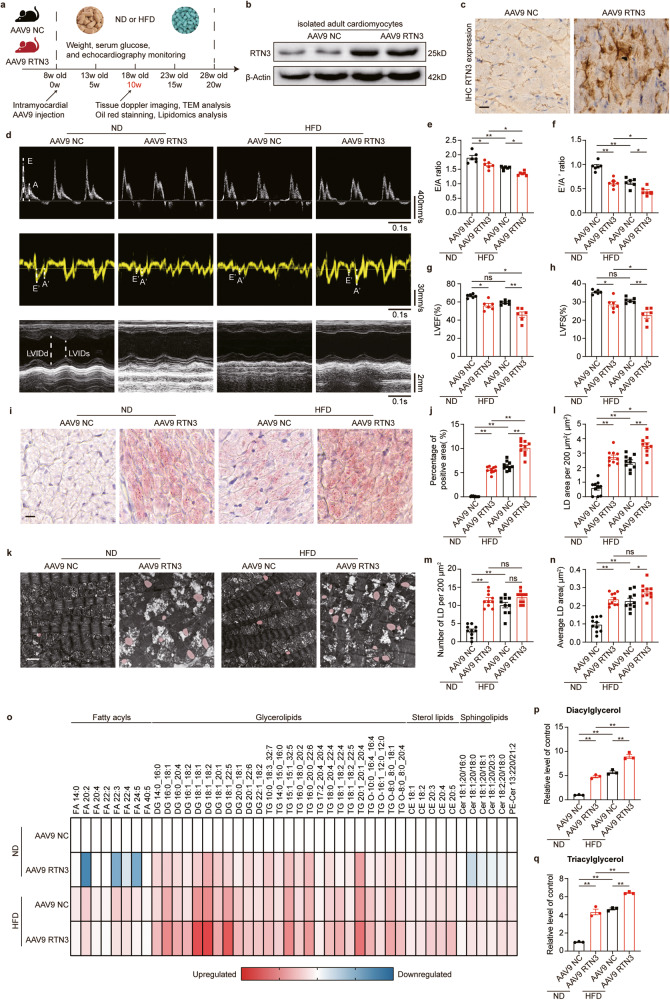
Cardiomyocyte-specific RTN3 overexpression reproduced HFD-induced cardiac dysfunction and myocardial lipid accumulation. **a** Diagram of the RTN3 overexpression construction and high fat diet protocol; **b** Representative western blotting images of RTN3 protein expression in isolated adult cardiomyocytes; **c** Representative immunohistochemical images indicating RTN3 protein level, scale bar = 40 μm; echocardiographic assessments were performed on AAV9 NC and AAV9 RTN3 mice after 10-week ND or HFD. *n* = 6 mice each group. Representative doppler, tissue doppler, and M-mode echocardiography images (**d**) and quantitative analysis of *E*/*A* ratio (**e**), *E*′/*A*′ ratio (**f**), LVEF (**g**), and LVFS (**h**); **i** Representative oil red staining images indicating intramyocardial lipid content, scale bar = 20 μm; **j** Quantitative analysis of positive area of oil red staining (*n* = 10 images each group); **k** Representative TEM images of myocardium, LDs were labeled as red. Scale bar = 2 μm; Quantitative analysis of LD area per 200 μm^2^ (**l**), LD number per 200 μm^2^ (**m**), and average LD area (**n**) (*n* = 10 images each group); **o** Heatmap of metabolic alterations organized by lipid class (*n* = 3 mice each group); Quantitative analysis of diacylglycerol (**p**) and triacylglycerol (**q**) (*n* = 3 mice each group). Data are expressed as Mean ± SEM. Differences are significant for *P < 0.05, **P < 0.01.

To further determine the role of RTN3 in cardiac lipid accumulation and dysfunction, we developed cardiomyocyte-specific RTN3-overexpressing mice via intramyocardial injection of adeno-associated virus 9 (AAV9) expressing RTN3 (defined as AAV9 RTN3). Mice injected with AAV9 NC were used as control mice (defined as AAV9 NC) (Fig. 2a). The successful development of RTN3-overexpressing mice was confirmed by western blotting and immunohistochemical (IHC) staining (Fig. 2b, c, Supplementary Fig. 2a, b). Compared with control mice, AAV9 RTN3 injection induced apparent cardiac diastolic and systolic function impairment in mice fed with ND, which phenocopied the phenomenon in mice fed with prolonged HFD (Fig. 2d–h, Supplementary Fig. 3, Supplementary Tables 2 and 3). As shown in Fig. 2d–h, similar to WT mice, AAV9-NC-injected mice exhibited unchanged cardiac function after 10 weeks of ND feeding, as demonstrated by preserved diastolic and systolic indicators. However, both cardiac diastolic and systolic functions in ND-fed AAV9-RTN3-injected mice were significantly reduced compared to those in control mice. Moreover, HFD feeding led to more severe impairment of cardiac function in AAV9-RTN3-injected mice (Fig. 2d–h). Collectively, these data suggested that RTN3 overexpression could mimic the deleterious effects of HFD on cardiac function. The effect of RTN3 overexpression on cardiac lipid accumulation was then determined. As shown in Supplementary Fig. 2c–h, body weight, 12 h fasting blood glucose, and serum lipids (including TG, CHO, HDL, and LDL) were not different between AAV9 RTN3 mice and AAV9 NC mice, indicating that cardiomyocyte-specific RTN3 overexpression did not affect systematic metabolism. However, a significant increase in the Oil Red O staining-positive area in the hearts of AAV9-RTN3-injected mice was observed (Fig. 2i, j), suggesting that RTN3-overexpressed mice developed cardiac lipid accumulation with or without exposure to HFD. TEM analysis also revealed that the number and area of LDs were obviously increased in the hearts of AAV9 RTN3 mice (Fig. 2k–n), indicating that RTN3 overexpression led to LD accumulation in the heart. Moreover, RTN3 overexpression significantly increased the proportion of large LDs (Supplementary Fig. 2i). These findings were further confirmed by lipidomic analysis of heart tissues across the four groups (Fig. 2o–q, Supplementary Fig. 4). We found that AAV9 RTN3 administration increased glycerolipids, including diacylglycerol and triacylglycerol, in both ND and HFD groups (Fig. 2p, q), suggesting a role for RTN3 in boosting the neutral lipids pool. There was also a decrease in fatty acid levels in the hearts of AAV9 RTN3 mice (e.g., FA 20:4 and FA 22:2, Supplementary Fig. 4b), which correlated with the increase in glycerolipids levels. Additional changes in other lipid classes were also observed. Results showed that overexpression of RTN3 induced increase in sterol lipids (Supplementary Fig. 4c, g), but did not alter the level of sphingolipids in the heart (Supplementary Fig. 4e, h). Collectively, overexpression of RTN3 led to significant intramyocardial lipid accumulation and cardiac dysfunction, which reproduced the phenotype caused by the HFD.

### Cardiomyocyte-specific RTN3 deletion prevented HFD-induced cardiac dysfunction and myocardial lipid accumulation

**Fig. 3:**
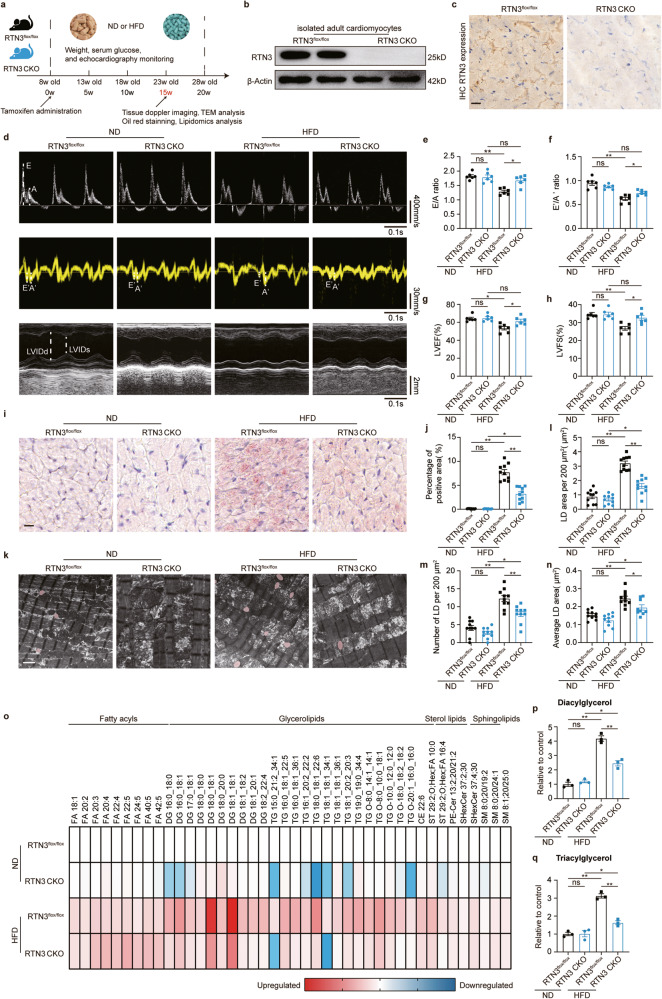
Cardiomyocyte-specific RTN3 deletion ameliorated HFD-induced cardiac dysfunction and myocardial lipid accumulation. **a** Diagram of the RTN3 knockout construction and high fat diet protocol; **b** Representative western blotting images of RTN3 protein expression in isolated adult cardiomyocytes; **c** Representative immunohistochemical images indicating RTN3 protein level, scale bar = 40 μm; **d–h** echocardiographic assessment was performed on RTN3 ^flox/flox^ and RTN3 CKO mice after 15-week ND or HFD. n = 6 mice each group. Representative doppler, tissue doppler, and M-mode echocardiography images (d) and quantitative analysis of *E*/*A* ratio (**e**), *E*′/*A*′ ratio (**f**), LVEF (**g**), and LVFS (**h**); **i** Representative oil red staining images indicating intramyocardial lipid content, scale bar = 20 μm; **j** Quantitative analysis of positive area of oil red staining (*n* = 10 images each group); **k** Representative TEM images of myocardium, LDs were labeled as red. Scale bar = 2 μm; Quantitative analysis of LD area per 200 μm^2^ (**l**), LD number per 200 μm^2^ (**m**), and average LD area (**n**) (*n* = 10 images each group); **o** Heatmap of metabolic alterations organized by lipid class (*n* = 3 mice each group); Quantitative analysis of diacylglycerol (**p**) and triacylglycerol (**q**) (*n* = 3 mice each group). Data are expressed as Mean ± SEM. Differences are significant for *P < 0.05, **P < 0.01.

Next, we generated cardiomyocyte-specific RTN3 knockout mice to determine whether RTN3-mediated cardiac lipid accumulation is the key factor in the development of HFD-induced cardiac dysfunction. RTN3^flox/flox^αMHC^Cre+^ mice were used as RTN3 CKO mice, and littermate RTN3^flox/flox^ mice were used as controls (Fig. 3a). Cre expression was induced by intraperitoneal injection of Tamoxifen, and then western blotting and IHC staining were used to confirm the cardiomyocyte-specific deletion of RTN3 (Fig. 3b, c, Supplementary Fig. 5a, b). RTN3 CKO and RTN3^flox/flox^ mice were fed with either ND or HFD for 20 weeks. First, multiple echocardiography was performed to determine the effect of RTN3 deletion on cardiac dysfunction. Consistent with our results in WT mice, 15- and 20-week HFD-fed RTN3^flox/flox^ mice developed significantly reduced diastolic and systolic function, as indicated by the decreased E/A ratio, E’/A’ ratio, LVEF, and LVFS (Fig. 3d–h, Supplementary Fig. 6, Supplementary Tables 4 and 5). However, HFD failed to induce cardiac dysfunction in RTN3 CKO mice, and both diastolic and systolic function of 15- and 20-week HFD-fed RTN3 CKO mice remained comparable to that of ND-fed mice (Fig. 3d–h, Supplementary Fig. 6). This suggests that RTN3 deletion protected against the deleterious effects of HFD on cardiac function. Next, we aimed to determine whether RTN3 deletion was sufficient to prevent cardiac lipid accumulation. As shown in Supplementary Fig. 5c–h, overall metabolic indicators, including body weight, 12 h fasting blood glucose, and serum lipids (including TG, CHO, HDL, and LDL), showed no significant difference between RTN3-CKO mice and RTN3^flox/flox^ mice. In contrast, compared with HFD-fed RTN3^flox/flox^ mice, the hearts of HFD-fed RTN3 CKO mice showed a reduction in the area positive for Oil Red O staining (Fig. 3i, j). TEM images also revealed fewer and smaller LDs in the hearts of RTN3 CKO mice than those in RTN3^flox/flox^ mice fed with HFD (Fig. 3k–n, Supplementary Fig. 5i), indicating that RTN3 CKO mice were resistant to HFD-induced myocardial lipid accumulation. Lipidomic analysis was performed to further investigate the role of RTN3 knockout in cardiac lipid metabolism (Fig. 3o–q, Supplementary Fig. 7). RTN3 deletion significantly reduced the levels of most glycerolipids. Specifically, the HFD-induced TG 15:0_21:2_34:1, TG 18:1_18:1_36:1, and TG 19:0_19:0_34:4 elevations returned to almost normal levels after RTN3 deletion (Supplementary Fig. 7d). In addition, HFD-fed RTN3 CKO mice displayed a reduction in sterol lipids (Supplementary Fig. 7c, g) while the sphingolipids amount remained unaffected by RTN3 deletion (Supplementary Fig. 7e, h). Considered together, these results suggest that RTN3 deletion prevents HFD-induced intramyocardial lipid accumulation and further protects against HFD-induced cardiac dysfunction.

### RTN3 was sufficient to induce LD biogenesis in cardiomyocytes

**Fig. 4:**
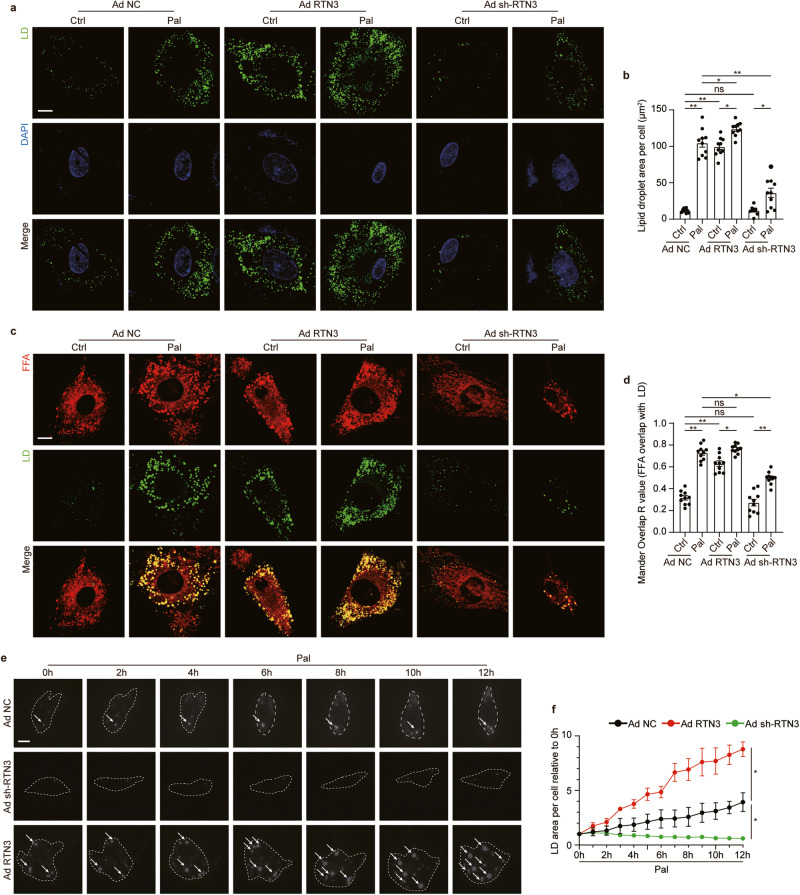
RTN3 was sufficient to induce LD biogenesis in cardiomyocytes. Representative fluorescence images indicating LDs labeled with Bodipy 493/503 and nucleus labeled with DAPI (**a**) and quantitative analysis of LDs area per cell (**b**) (*n* = 10 images each group), scale bar = 10 μm; Representative fluorescence images indicating FFA labeled with Bodipy 558/568 C12 and LDs labeled with Bodipy 493/503 (**c**) and quantitative analysis of localization of FFA with LDs quantified with Mandar overlap R value analysis (**d**) (*n* = 10 images each group), scale bar = 10 μm; **e** Time-lapse montage of LDs biogenesis in live cells, scale bar = 10 mm; **f** Quantitative analysis of LD area (*n* = 10 images each group). Data are expressed as Mean ± SEM. Differences are significant for *P < 0.05, **P < 0.01.

The linkage between RTN3 and myocardial lipid accumulation were further investigated in vitro in primary neonatal rat ventricular cardiomyocytes (NRVCs). Given that long-chain fatty acid is a major fuel of the heart [5], palmitate (Pal) was employed to mimic the effect of HFD on cardiomyocytes. As shown in Supplementary Fig. 8a–c, with the increasing dose of Pal, dose-dependent increases in LDs content and RTN3 expression were observed in NRVCs, both of which reached the maximum at the dose of 500 μM Pal. Therefore, 500 μM Pal was employed in our subsequent in-vitro study. Consistent with results in vivo, Pal treatment resulted in a significant increase of RTN3 expression in NRVCs (Supplementary Fig. 8c). Adenovirus (Ad) was then used to upregulate or downregulate RTN3 in NRVCs (Supplementary Fig. 8d). Infection with Ad RTN3, with or without Pal, increased the pool of LDs and phenocopied the effects of Pal stress (Fig. 4a, b). In contrast, Pal-induced LD accumulation was blocked by the administration of Ad sh-RTN3 (Fig. 4a, b), indicating that RTN3 could affect LD content in cardiomyocytes.

Due to the characteristic C-terminal hairpin transmembrane domain, RTN3 resides in the tubular ER, where LD biogenesis occurs. Based on this fact, we hypothesized that the effect of RTN3 on LD content could be attributed to its direct effect on LD biogenesis in the ER. To test this hypothesis, we employed red fluorescence-labeled FA to visualize its track upon being taken up by NRVCs (Fig. 4c). Twelve hours after administration of labeled FA, cells infected with Ad RTN3 displayed markedly increased FA-LD colocalization (Mander Overlap R value from 0.32 to 0.63, Fig. 4c, d), similar to the effect of Pal treatment. This increase in colocalization was absent in NRVCs infected with Ad sh-RTN3 with or without Pal (Fig. 4c, d). To further confirm this observation, we constructed a time-lapse montage of LDs in NRVCs during Pal stress (Fig. 4e, Supplementary Video 1–3). Quantification analysis revealed that Ad RTN3 administration enhanced LD biogenesis in NRVCs, as evidenced by a more rapid increase in the LD area, and an opposite trend was observed in NRVCs infected with Ad sh-RTN3 (Fig. 4f). These results indicated that RTN3 was adequate to induce LD biogenesis and affect LD content in NRVCs, while RTN3 suppression led to defective LD biogenesis.

Despite LD content in cardiomyocytes is mainly dependent on LD biogenesis, it was also affected by other lipid metabolic processes including FAs oxidation in mitochondria and FAs uptake. Therefore, FAs uptake and oxidation were determined in cardiomyocytes with RTN3 overexpression or ablation. As shown in Supplementary Fig. 8e, both RTN3 overexpression and knockdown did not influence the expression of CD36, the main mediator for FAs uptake in cardiomyocytes, suggesting that FA uptake was not affected by RTN3. For determination of FAs oxidation, FAs, mitochondria and LDs were labeled in cardiomyocytes respectively. As shown in Supplementary Fig. 8f, g and Fig. 4e, f, in normal-cultured cardiomyocytes, FAs predominantly positioned themselves in mitochondria, with little FAs localized in the LDs. In contrast, RTN3 overexpression or Pal incubation led to a significant redistribution of FAs from mitochondria to LDs, while RTN3 knockdown prevented the deposition of FAs in LDs induced by Pal treatment. Taken together, these data suggested that RTN3-drived LDs biogenesis by regulating the subcellular orientation of FAs, but not by promoting FAs uptake.

### RTN3 interacted with FABP5 to promote LD biogenesis on ER

**Fig. 5:**
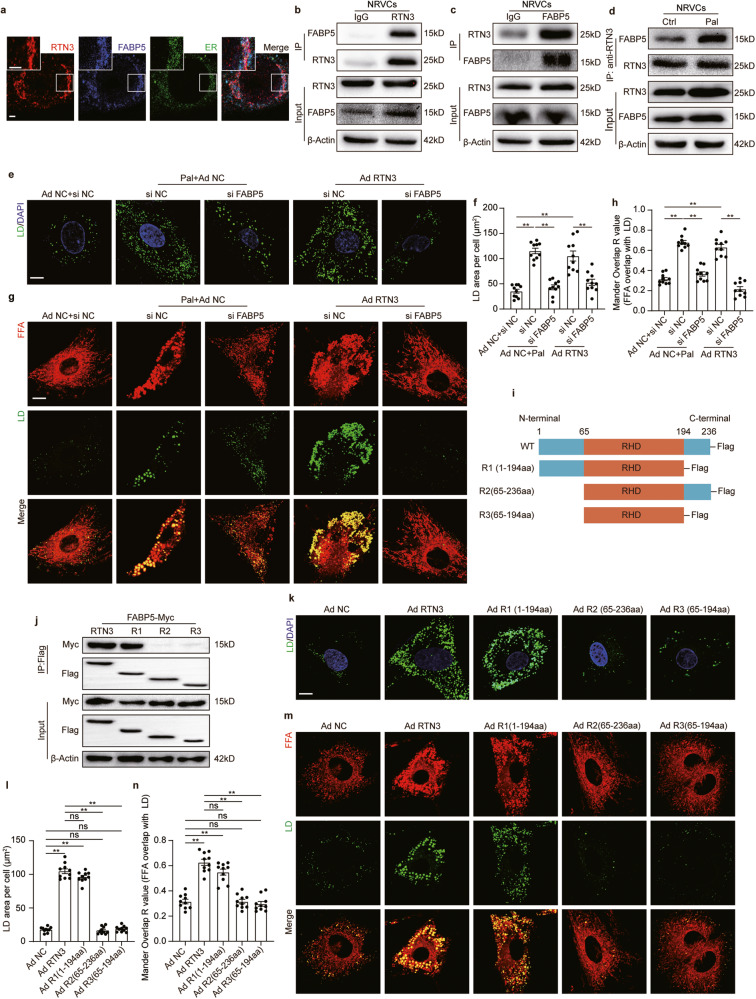
RTN3 formed a protein complex with FABP5 to promote LD biogenesis. **a** Representative immunofluorescence images indicating RTN3, FABP5, and ER, scale bar = 2.5 μm; Representative western blotting images of IP assay using anti-RTN3 (**b**) or anti-FABP5 (**c**) to determine the interaction of RTN3 and FABP5 in NRVCs; **d** Representative images of IP assay using anti-RTN3, NRVCs were used to perform the IP assay; Representative fluorescence images indicating LDs labeled with Bodipy 493/503 and nucleus labeled with DAPI (**e**) and quantitative analysis of LDs area per cell (**f**) (*n* = 10 images each group), scale bar = 10 μm; Representative fluorescence images indicating FFA labeled with Bodipy 558/568 C12 and LDs labeled with Bodipy 493/503 (**g**) and quantitative analysis of localization of FFA with LDs quantified with Mandar overlap *R* value analysis (**h**) (*n* = 10 images each group), scale bar = 10 μm; **i** Schematic diagrams of wild type and truncated forms of RTN3; **j** Representative western blotting images of IP assay using anti-Flag; Representative fluorescence images indicating LDs labeled with Bodipy 493/503 and nucleus labeled with DAPI in NRVCs infected with truncated RTN3 (**k**) and quantitative analysis of LDs area per cell (**l**) (*n* = 10 images each group), scale bar = 10 μm; Representative fluorescence images indicating FFA labeled with Bodipy 558/568 C12 and LDs labeled with Bodipy 493/503 in NRVCs infected with truncated RTN3 (**m**) and quantitative analysis of localization of FFA with LDs quantified with Mandar overlap *R* value analysis (**n**) (*n* = 10 images each group), scale bar=10μm. Data are expressed as Mean ± SEM. Differences are significant for **P < 0.01.

To further explore the mechanism by which RTN3 influenced the orientation of FAs and promoted LDs biogenesis, Co-immunoprecipitation (Co-IP) coupled with liquid chromatography-tandem mass spectrometry (LC-MS/MS) analysis was used to identify the interaction partners of RTN3 in cardiomyocytes. Fatty Acid Binding Protein 5 (FABP5), a famous intracellular carrier for long-chain FAs, was found in top-ranked RTN3-binding proteins and was selected for further analysis (Supplementary Table 6). Western blotting results demonstrated that FABP5 expression was significantly increased in the hearts and isolated adult cardiomyocytes of HFD feeding mice (Supplementary Fig. 9a–d). Moreover, overexpression or deletion of RTN3 expression did not affect FABP5 level both in vivo and in vitro (Supplementary Fig. 9e–g). Several additional experiments were performed to confirm the physical interaction between RTN3 and FABP5. Immunofluorescence analysis revealed that RTN3 colocalized well with FABP5 in cardiomyocytes (Fig. 5a). Co-IP using antibodies against either RTN3 or FABP5 also demonstrated their interaction (Fig. 5b, c). Moreover, it was discovered that the level of RTN3-FABP5 complex was increased in NRVCs with Pal treatment compared to control ones (Fig. 5d). These results were further confirmed in the cardiac tissues of mice (Supplementary Fig. 9h–j). Based on the RTN3-FABP5 interaction, we propose that this interaction may form the structural basis for the directional transport of FAs from the cytoplasm to the ER to accomplish LD biogenesis, and silencing any one of them would disturb the structure and inhibit LD biogenesis. To test this hypothesis, we first silenced FABP5 using siRNAs. Western blotting results suggested that silencing of FABP5 did not alter the expression of RTN3 in cardiomyocytes (Supplementary Fig. 9k). LD staining results showed that silencing FABP5 blunted LD boosting induced by RTN3 overexpression (Fig. 5e, f). Moreover, as described previously, Ad RTN3 administration enhanced the channeling of FA into LDs; however, such effects were attenuated by silencing FABP5 (Fig. 5g, h).

To further identify the interaction site of RTN3 and FABP5, myc-tagged FABP5 and flag-tagged three truncated forms of RTN3, namely R1 (1st–194th amino acids, lacking C-termini), R2 (65th–236th amino acids, lacking N-termini), and R3 (65th–194th amino acids, lacking C- and N- termini), were constructed and introduced into 293T cells (Fig. 5i). Co-IP results demonstrated that only RTN3 with full length and R1 could interacted with FABP5, while R2 and R3 failed in establishing interaction with FABP5 (Fig. 5j), suggesting that the 1st–65th amino acids of RTN3 were indispensable for the interaction between RTN3 and FABP5. Furthermore, R1 induced a parallel effect on LD content and FAs channeling when compared with full length RTN3 (Fig. 5k–n), while R2 and R3 were incapable to exert such effects. These data suggested that 1st-65th amino acids of RTN3 were critical for RTN3-FABP5 interaction and regulating LD biogenesis. Collectively, these results demonstrate that RTN3 physically and functionally coupled with FABP5, facilitating LD biogenesis and promoting lipid accumulation in cardiomyocytes.

### RTN3-induced LDs biogenesis is dependent on diacylglycerol acyltransferase 2 (DGAT2) but not diacylglycerol acyltransferase 1 (DGAT1)

DGAT1 and DGAT2 are two key enzymes for the synthesis of neutral lipids, which is also essential for initiating LD biogenesis [12]. To further reveal the molecular details involved in RTN3-induced LD biogenesis, the effect of DGAT1 and DGAT2 was investigated. Results revealed that both in the hearts and in isolated adult cardiomyocytes, the expression of DGAT1 and DGAT2 were significantly upregulated in HFD feeding mice (Supplementary Fig. 10a–e). However, overexpression or deletion of RTN3 would not induce statistically different changes of DGAT1 and DGAT2 protein level (Supplementary Fig. 10f–h), suggesting RTN3 did not affect the expression of them. Conversely, silencing DGAT1 nor DGAT2 also did not alter the expression of RTN3 (Supplementary Fig. 10i). To elucidate the functional linkage between RTN3 and these enzymes, the LD amount was investigated in RTN3-overexpressed cardiomyocytes transfected with siDGAT1 or siDGAT2 (Supplementary Fig. 10j, k). Interestingly, DGAT2 knockdown induced a stronger inhibition on RTN3-induced LD accumulation compared with DGAT1 knockdown. Moreover, siDGAT2 alone induced a comparable effect to that of simultaneous knockdown of DGAT1 and DGAT2. These results indicate that DGAT2 is preferentially required in RTN3-induced LD biogenesis. Immunofluorescence study was conducted to further confirm the pivotal role of DGAT2 in RTN3-induced LD biogenesis. As shown in Supplementary Fig. 10l, when LD biogenesis was activated either by Pal stress or RTN3 overexpression, DAGT2 (shown as blue) and RTN3 (shown as red) formed a ring-like structure around LDs (shown as green). Taken together, these data suggested a complex formed by RTN3/FABP5 interaction and DGAT2, which provided both structural and catalytic basis for neutral lipids synthesis and LD biogenesis.

### C/EBPα directly regulated the transcription of RTN3

**Fig. 6:**
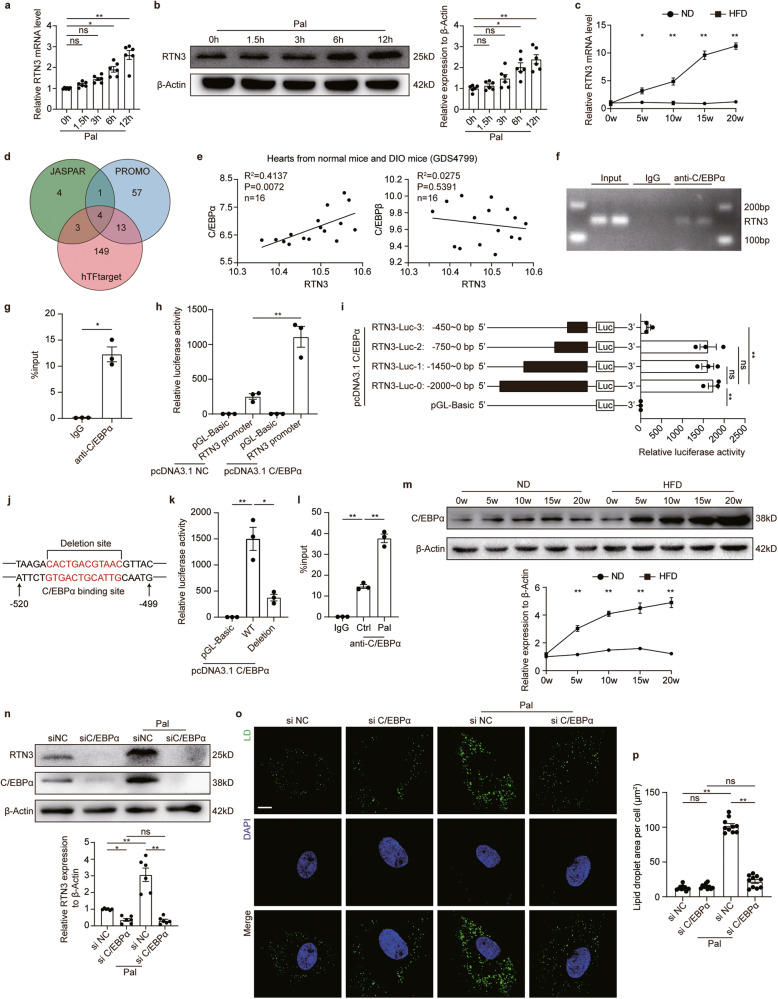
C/EBPα regulated the transcription of RTN3. **a** Quantitative analysis of RTN3 mRNA level in NRVCs treated with Pal (*n* = 6 wells each group); **b** Representative western blotting images and quantitative analysis of RTN3 protein level (*n* = 6 wells each group); **c** Quantitative analysis of RTN3 mRNA level in the hearts of ND- and HFD-fed mice (*n* = 6 mice each group); **d** Venn diagram showing transcription factors identified with three databases; **e** Correlation between the mRNA level of C/EBPα and C/EBPβ and RTN3 based on a public data set (GSE40722); **f** ChIP analysis for C/EBPα binding to the RTN3 promoter in NRVCs; **g** q-PCR analysis of DNA obtained from ChIP (*n* = 3 each group); **h** Relative luciferase activity of cells infected with different plasmids (*n* = 3 wells each group); **i** Relative luciferase activity of cells infected with plasmids expressing truncated RTN3 promotor region and C/EBPα (*n* = 3 wells each group); **j** Schematic diagrams of wild type and deleted forms of RTN3 promotor region; **k** Relative luciferase activity of cells infected with plasmids expressing wild type and deleted forms of RTN3 promotor region and C/EBPα (*n* = 3 wells each group); **l** q-PCR analysis of DNA obtained from ChIP with or without palmitate treatment; **m** Representative western blotting images and quantitative analysis of C/EBPα protein level (*n* = 6 mice each group); **n** Representative western blotting images and quantitative analysis of RTN3 protein level (*n* = 6 wells each group); Representative fluorescence images indicating LDs labeled with Bodipy 493/503 and nucleus labeled with DAPI (**o**) and quantitative analysis of LDs area per cell (**p**) (*n* = 10 images each group), scale bar = 10 μm. Data are expressed as Mean ± SEM. Differences are significant for *P < 0.05, **P < 0.01.

Above results showed that RTN3 expression was upregulated in the hearts of HFD-fed mice. We then investigated the mechanism underlying the upregulation of RTN3. First, NRVCs were treated with Pal for different durations, and the mRNA and protein levels of RTN3 were assessed. RTN3 mRNA levels gradually increased with Pal treatment (Fig. 6a). Western blotting results demonstrated the same trend for RTN3 protein (Fig. 6b). Moreover, results from the hearts of HFD-fed mice also displayed the consistency between RTN3 mRNA and protein (Fig. 6c), suggesting that the expression level of RTN3 was regulated at the transcriptional level. Based on that, we examined the transcriptional factors of RTN3 by querying the JASPAR, PROMO, and hTFtarget databases. Four overlapping transcription factors of RTN3, namely, C/EBPα, C/EBPβ, nuclear factor 1C (NF1C), and transcriptional repressor protein YY1, were identified for further analysis (Fig. 6d). Among these, the C/EBP family is reported to be involved in adipocyte differentiation and maturation, and multiple genes related to lipid metabolism are transcriptionally regulated by the C/EBP family. Therefore, we investigated the correlation of C/EBP α/β with RTN3 using data derived from the Gene Expression Omnibus (GEO) database (GDS4799). Pearson’s correlation analysis revealed a significant positive correlation between C/EBPα and RTN3, whereas such a correlation was absent between C/EBPβ and RTN3 (Fig. 6e). Collectively, C/EBPα was selected as a possible transcription factor of RTN3 for further validation and analysis. Chromatin immunoprecipitation (ChIP)-quantitative PCR analysis validated the direct binding of C/EBPα to the promoter region of RTN3 (Fig. 6f, g). Further dual-luciferase reporter assay demonstrated C/EBPα could directly regulate the transcription of RTN3 (Fig. 6h). We then constructed plasmids containing fragments of RTN3 promotor region to identify the specific binding sites of C/EBPα. As shown in Fig. 6i, the relative luciferase activity was significantly decreased in cells transfected with RTN3-Luc-3 (−450 to 0 bp), while the luciferase activity was comparable between cells transfected with RTN3-Luc-2 (−750 to 0 bp) and RTN3-Luc-0 (−2000 to 0 bp), indicating the C/EBPα regulatory site was within the −450 to −750 bp of RTN3 promotor region. Then, the specific C/EBPα binding site was predicted by sequencing analysis, and the possible sequence “GTGACTGCATTG” located in the region from −504 bp to −515 bp was found (Fig. 6j). Further experiments demonstrated that cells transfected with RTN3 promoter region with “GTGACTGCATTG” sequence deletion exhibited obviously decreased luciferase activity compared to wild type promoter region (Fig. 6k), revealing the “GTGACTGCATTG” sequence was the site within RTN3 promoter region targeted by C/EBPα. We then investigated the relation between C/EBPα and RTN3 under palmitate treatment. CHIP-qPCR results suggested that the interaction between C/EBPα and RTN3 promoter region was increased under palmitate stress (Fig. 6l). Besides, the expression of C/EBPα was upregulated in the hearts of HFD feeding mice and cardiomyocytes treated with palmitate (Fig. 6m, n). Knockdown of C/EBPα significantly suppressed Pal-induced upregulation of RTN3 (Fig. 6n), suggesting that C/EBPα is a positive transcription factor of RTN3 and is responsible for Pal-induced RTN3 upregulation. Additionally, C/EBPα knockdown significantly inhibited Pal-induced LD accumulation (Fig. 6o, p). Considered together, these data suggest that the increased expression of C/EBPα is responsible for lipid overload-induced RTN3 activation. C/EBPα directly binds to the promoter region of RTN3 and positively regulates its expression.

### RTN3-mediated lipid accumulation in heart tissue from obese patients

**Fig. 7:**
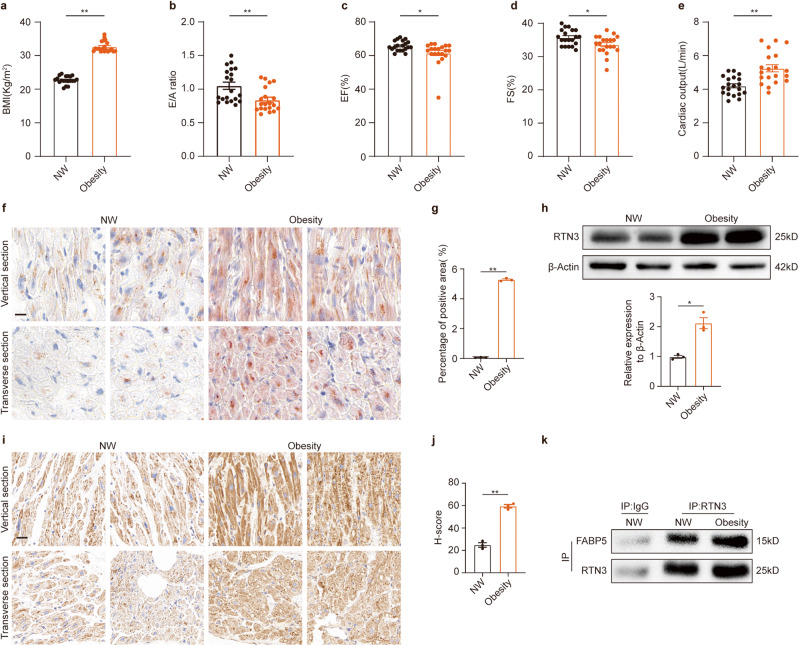
RTN3-mediated lipid accumulation in heart tissue from obese patients. Quantitative analysis of BMI (**a**), E/A ratio (**b**), LVEF (**c**), LVFS (**d**), and cardiac output (**e**) in obese patients and NW individuals (*n* = 20 individuals each group); representative oil red staining images indicating intramyocardial lipid content (**f**) and quantitative analysis of positive area of oil red staining (**g**) (*n* = 3 individuals each group), scale bar = 20 μm. **h** Representative western blotting images and quantitative analysis of RTN3 protein level (*n* = 3 individuals each group); Representative immunohistochemical images indicating RTN3 protein level (**i**) and quantitative analysis of H-score of immunohistochemical staining images (**j**) (*n* = 3 individuals each group), scale bar = 30 μm; **k** Representative western blotting images of IP assay using different antibody to determine the interaction of RTN3 and FABP5. Data are expressed as Mean ± SEM. Differences are significant for *P < 0.05, **P < 0.01.

To translate our insights into the context of human diseases, RTN3-mediated lipid accumulation was examined in right atrial tissues obtained from obese patients and individuals with BMI between 18.5 kg/m^2^ and 24.9 kg/m^2^ (defined as NW). As shown in Fig. 7a, the BMI of obese patients was significantly higher than that of nonobese individuals. Consistent with the diastolic dysfunction observed in HFD-fed mice, the E/A ratio in obese patients was decreased (Fig. 7b). A statistically significant decrease in ejection fraction and fractional shortening was also observed between the two groups (Fig. 7c, d), demonstrating the adverse effect of obesity on cardiac systolic function. In addition, obese patients had higher cardiac output and left atrial diameter (Fig. 7e, Supplementary Table 7). Oil Red O staining was performed to quantify myocardial lipids in obese patients (Fig. 7f). Quantitative analysis demonstrated an obvious increase in the positive area in obese hearts (Fig. 7g), indicating cardiac lipid accumulation in obese individuals. Consistent with the upregulation of RTN3 observed in HFD-fed mice, western blotting and IHC results revealed that the protein level of RTN3 was dramatically increased in the hearts of obese patients (Fig. 7h–j). Moreover, we performed co-IP experiments to validate the RTN3-FABP5 interaction in human hearts. We found that RTN3 directly interacted with FABP5, and this interaction was enhanced in the myocardia of patients with obesity (Fig. 7k).

## Discussion

**Fig. 8:**
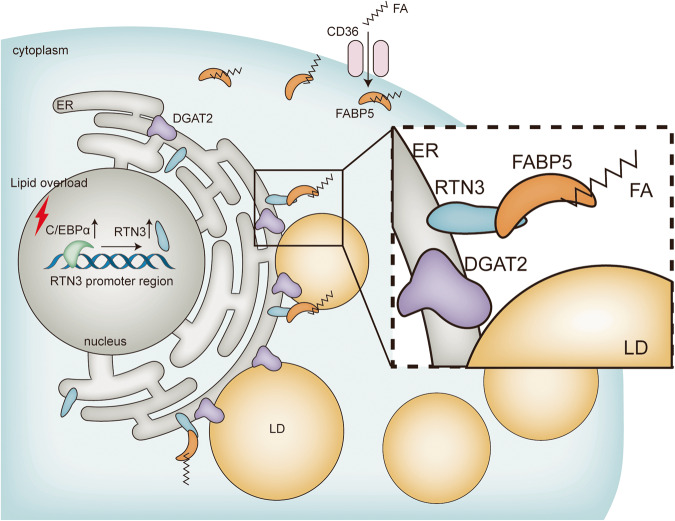
Schematic figure illustrating that the RTN3-mediated LD biogenesis in cardiomyocytes. Under lipid overload conditions, C/EBPα was upregulated, which then activated the transcription of RTN3. The increased RTN3 interacted with FABP5 in ER membrane and facilitated the transportation of FA to ER, thereby promoting LDs biogenesis. Moreover, DGAT2 was the key enzyme responsible for this process, which colocalized with RTN3 around LD. The pathologically activated RTN3-mediated LD biogenesis led to LD increase in cardiomyocytes and myocardial lipid accumulation. C/EBPα CCAAT/enhancer binding protein α, RTN3 reticulon 3, ER endoplasmic reticulum, DGAT2 diacylgycerol acyltransferase 2, FABP5 fatty acid binding protein 5, FA fatty acids, LD lipid droplet.

This study revealed a novel mechanism contributing to LD biogenesis. In cardiomyocytes, ER-localized RTN3 directly interacts with FABP5 and facilitates FABP5-mediated FA directional transport to the ER, thereby promoting DGAT2-dependent LD biogenesis. Under lipid overload conditions, upregulated RTN3 forms more complex with FABP5 and accelerates LD biogenesis in cardiomyocytes, leading to excessive LD accumulation and cardiac dysfunction. In contrast, genetic ablation of RTN3 significantly suppresses LD accumulation in the myocardium and protects against lipid overload-induced cardiac dysfunction. Moreover, the upregulation of RTN3 in lipid-overloaded hearts was induced by the increased expression of C/EBPα, which positively regulates RTN3 transcription by directly binding to the “GTGACTGCATTG” sequence (−504 bp to −515 bp) within RTN3 promoter region (Fig. 8).

LDs are key organelles that store lipids and supply FAs for energy generation, playing important roles in lipid metabolism and energy homeostasis [18]. Although the role of LDs in the heart is still debated, excessive cardiac LD accumulation in hyperlipidemic states is regarded as a negative factor for cardiac function. In this study, we found that LD accumulation in the heart, caused by either HFD or RTN3 overexpression, could induce diastolic and systolic function impairment, indicating a negative effect of LD in the heart. Consistent with our results, genetically modified mice with increased cardiac LD also develop various heart diseases, including cardiac hypertrophy, dilated cardiomyopathy, heart failure, and even death [9, 19, 20]. Mechanistically, studies reported that increased cardiac LD amount was associated with disordered architecture of cardiomyocytes, impaired mitochondrial dynamics, and increased cell death, which might account for the detrimental cardiac effects induced by LD accumulation [9, 19, 21, 22]. However, other studies have proposed that LD may act as a safe reservoir that sequesters toxic lipid intermediates into LDs and protects the heart against lipotoxicity [23]. For instance, during prolonged starvation, LDs can accommodate excessive acylcarnitine and prevent mitochondrial damage [24]. This complexity and disagreement may arise from the different metabolic states of the cells. LDs induced by nutrient deprivation tend to accommodate autophagy-liberated FAs and then be lipolytically degraded to sustain the energy supply. In contrast, LDs induced by prolonged lipid overloading are more resistant to lipolytic degradation and tend to accumulate in cardiomyocytes. In this study, silencing of RTN3 did not alter the amount of LD or cardiac function of ND-fed mice, indicating that RTN3-mediated LD biogenesis participated less in cardiac energy generation. Apparently, in the hearts of the continuous HFD-fed mice employed in our study, RTN3-mediated cardiac LDs biogenesis was a response to elevated circulating levels of FAs and was prone to deposition. These data suggested that RTN3-driven LDs biogenesis is detrimental and plays a pathogenic role in the development of HFD-induced cardiac dysfunction.

LDs biogenesis is a complex process, and the detailed mechanisms are still being investigated. The current model for LDs biogenesis comprises multiple steps, including neutral lipids synthesis, LD budding, and LD growth and maturation, and neutral lipids synthesis, the crucial initiating step, occurs primarily in ER [12]. During this initial step, FAs are transported to the ER and esterified to glycerol to produce TG [12]. However, it remains poorly understood how FA transportation is oriented and regulated. In this study, we revealed that the ER-resident protein RTN3 directly binds with the FAs carrier FABP5, which provides an anchorage mechanism that facilitates FAs directional transport and LDs biogenesis. The evidence can be summarized as follows: First, RTN3 physically interacts with FABP5 at the ER membrane, which was confirmed by co-IP assays and further immunofluorescence. Second, disruption of this interaction by silencing RTN3 or FABP5 impaired LDs biogenesis, while enhancement of this interaction through RTN3 overexpression or Pal treatment remarkably increased LD number and volume. Moreover, our study proved that RTN3/FABP5 promoted LDs biogenesis in a DGAT2-dependent manner. Specifically, the knockdown of DGAT2 greatly impaired RTN3-induced LDs biogenesis in cardiomyocytes. DGAT1 and DGAT2 belong to the same protein family and participate in TG synthesis. However, studies have shown that these two enzymes have distinct functions and subcellular distribution [25]. DGAT2-dependent LDs are larger in volume and responsible for energy storage and supply, while DGAT1 mediates the formation of small LDs under stress [26]. DAGT2 deletion in the mouse liver leads to the near absence of TG [27], which concurs with our results in cardiomyocytes, suggesting the dominant role of DGAT2 in controlling lipid storage. Collectively, our study findings revealed that RTN3 functioned as an anchor for recruiting the FAs carrier, initiating substrate, and key enzyme for TG synthesis, which constructed a microenvironment for LD biogenesis in the ER. Our results represent a further step in revealing the details of neutral lipid synthesis and shed fresh light on LDs biogenesis regulation in lipid-overloaded cardiomyocytes.

RTN3 has been implicated in diverse functions in both physiological and pathological processes, including inhibition of amyloid-beta production in the nervous system [28], activation of P53 in hepatocellular carcinoma [16], and elimination of misfolded insulin in the pancreas [29]. However, the role of RTN3 in cardiovascular disease has not yet been investigated. In this study, we first identified RTN3 as a novel mediator of the development of HFD-induced cardiac lipid accumulation. RTN3 promoted LDs biogenesis in cardiomyocytes, as corroborated by the higher rate of increase and greater numbers of LD observed with live cell imaging. RTN3 inhibition effectively prevented lipid overload-induced LD accumulation. Consistent with our findings, a previous study demonstrated that RTN3 could promote lipid accumulation in adipocytes and lead to systematic obesity. These data suggest that RTN3 is a promoter of lipid accumulation in both lipid consumption and storage organs. In addition to promoting LDs biogenesis by interacting with FABP5, RTN3 was also reported to inhibit amyloid-beta production by interacting with beta-amyloid converting enzyme 1 (BACE1) and to facilitate endosome maturation by interacting with Rab9a, indicating that RTN3 may play an indispensable role in assisting ER function by interacting with ER-target proteins. Moreover, RTN3 serves distinct functions through distinct structural domains, with 1001st–1003rd amino acid domain interacting with BACE1 [28] and 844th–1032nd amino acids maintaining ER tubular structure [30]. Our study further indicates that the function of RTN3 in LDs biogenesis relies on its 1st–65th amino acids, which have not yet been defined. Data showed that fragment of RTN3 lacking 1st to 65th amino acids was unable to interact with FABP5. Moreover, a previous study also observed that deletion of 66th to 843rd amino acids in RTN3 did not affect its function in inducing LDs biogenesis [15]. To the best of our knowledge, we provide the first evidence suggesting that RTN3 is involved in the development of cardiovascular diseases and further apprises our current understanding of its functional domains.

The role of C/EBPα in adipocyte differentiation and maturation has been extensively studied. Gene ontology analysis of C/EBPα binding genes showed substantial enrichment of genes involved in lipid metabolism, over 60% of which overlapped with peroxisome proliferator-activated receptor γ (PPARγ) target locations [31]. C/EBPα is activated during adipogenesis; it regulates the transcription of a series of genes, including free fatty acid receptor 2 (Ffar2) and long-chain fatty acid CoA ligase 1 (Acsl1), to promote lipid storage [31, 32]. In this study, we performed ChIP analysis and dual-luciferase reporter assay to determine whether C/EBPα directly binds to the promoter region of RTN3 to regulate its transcription positively. Under lipid overload conditions, C/EBPα is significantly upregulated, leading to increased expression of RTN3. In contrast, C/EBPα silencing prevented RTN3 upregulation and further inhibited Pal-induced LD biogenesis in cardiomyocytes. The pattern by which C/EBPα modulates RTN3 under lipid overload conditions is in accordance with its proposed role of regulating other lipid metabolism-related genes, including CD36 and pyruvate dehydrogenase kinase 4 (PDK4) [31].

As for clinical translation, our study observed excess intramyocardial lipid accumulation in obese patients accompanied by diastolic function impairment. Consistent with our results, previous studies have also reported obesity-related cardiac dysfunction and myocardial lipid accumulation [7]. Moreover, we demonstrated that RTN3 protein levels increased significantly in the myocardium of patients with obesity. The interaction between RTN3 and FABP5 was also found in human cardiac tissue, consistent with the results obtained in animals and cardiomyocytes. These translational results suggest that our study has good clinical utility and offers potential for improving clinical therapies targeting obesity-related cardiac dysfunction.

Our study has some limitations. In terms of lipid metabolism, the involvement of calcium signaling in lipolysis and lipid storage has been suggested [33, 34]. Recent studies have reported that the function of RTN3 in physiological and pathological conditions is also modulated by ER calcium flux [14, 16]. Considering the key role of calcium signals in sustaining cardiac functional and metabolic features, there may be a more complex network among RTN3, calcium flux, and LDs biogenesis in the ER of cardiomyocytes. Further studies are required to confirm this hypothesis. Additionally, for ethical reasons, the human myocardial tissue used in this study was collected from the atrium but not from the ventricle. It would be more appropriate if RTN3 expression could be assessed in more human ventricular samples. Correlation analysis of RTN3 expression, cardiac function, and myocardial LD content in patients would provide more robust evidence to strengthen our findings. Despite these limitations, our study revealed a novel mechanism contributing to LD biogenesis in cardiomyocytes. These findings advance our current understanding of the pathophysiology of obesity-induced myocardial lipid accumulation and cardiac dysfunction and lay the groundwork for novel strategies to treat cardiac dysfunction in obese patients.

## Materials and methods

### Animals

All animal experiments were approved by Air Force Medical University Animal Use and Care Committee and were performed in accordance with the Guide for the Care and Use of Laboratory Animals. To avoid the interference of hormonal change, only male mice were used in this study. C57BL/6J mice were provided by the animal center of Air Force Medical University as wild-type (WT) mice. All mice were maintained with controlled environmental conditions of temperature (22 ± 0.5 °C), humidity (60 ± 5%) and lighting (12 h light/12 h dark cycle) and had free access to either normal diet (ND, 10 kcal% fat, Research Diets # D12450J) or high fat diet (HFD, 60 kcal% fat, Research Diets # D12492) and water.

### Conditional cardiac-specific RTN3 knockout mice

RTN3^flox/flox^ and αMHC-MerCreMer mice (C57BL/6J background) were generated by Shanghai Model Organisms Center, Inc via the CRISPR/Cas9 technology. Conditional cardiac-specific RTN3 knockout mice (RTN3^flox/flox^αMHC^Cre+^, defined as RTN3 CKO mice) were generated by crossing RTN3^flox/flox^ mice with αMHC-MerCreMer mice, and littermate RTN3^flox/flox^ mice were used as control ones. Tamoxifen was dissolved in corn oil (20 mg/ml) and intraperitoneally injected (75 mg/kg body weight) for 5 consecutive days to induce RTN3 knockout at indicated time point. Timeline of further analysis were described in Fig. 3a.

### Intramyocardial adeno-associated virus 9(AAV9) injection

Cardiac-specific AAV9 vectors expressing RTN3-3xflag (AAV9 RTN3) and empty adenoviral vectors (AAV9 NC) were constructed by Shanghai Genechem, Inc. Cardiac-specific RTN3 overexpression mice (defined as AAV9 RTN3 mice) were generated by intramyocardial injection of AAV9 RTN3, and littermate mice were used as control ones after intramyocardial injection of negative control AAV9 (defined as AAV9 NC mice). For the intramyocardial AAV9 injection, mice were anesthetized with 2% isoflurane. The chest skin was shaved and the chest was open to expose heart. 5 × 10^10^ μg AAV9 RTN3 or AAV9 NC were evenly injected into 4 sites of heart. Timeline of further analysis were described in Fig. 2a.

### Assessment of body weight and blood glucose in mice

Body weight and 12 h fasting blood glucose were measured in all mice every 5 weeks. Before measurement, mice were fasted for 12 h but were free to water. Blood glucose was measured through the tail vein (Glucometer 580, Yuwell, China). Six mice were used per group for body weight and 12 h fasting blood glucose analysis.

### Echocardiography

Cardiac echocardiography was conducted with VEVO 3100 echocardiography system (VisualSonics Inc., Toronto, Canada) as previously described [35]. Heart rate was monitored throughout with a real time ECG monitoring equipment. The amount of isoflurane was adjusted to maintain heart rate within the range of 400–450 bpm. M-mode echocardiography was conducted to record and assess left ventricular ejection fraction (LVEF) and left ventricular fractional shortening (LVFS). Doppler echocardiography was conducted to record and assess diastolic trans-mitral blood flow velocities for peak early (E) and late (A) fillings. All echocardiographic measurements and images were analyzed using VEVO 3100 software. Six mice were used per group for echocardiographic index analysis. Images representing average level of corresponding group were chosen as representative ones.

### Tissue harvesting

Mice were euthanized by CO_2_. For blood sample, the neck skin was cut open and carotid artery was exposed by blunt dissection. The blood sample was immediately collected upon carotid artery was cut. For heart tissue sample, the chest was cut open and heart was exposed and harvested by cutting the great vessels. Upon harvested, the heart was immediately placed in pre-cooled PBS and washed. Then the heart was weighed, divided and fixed or snap-frozen in liquid nitrogen for further analysis. The TL was measured. Six mice were used per group for HW/TL analysis.

### Lipidomics

Lipidomics was conducted as previously described [36]. Left ventricular tissue was used for lipidomics analysis. Upon harvested, the heart tissue was put in liquid nitrogen until metabolites extraction. Lipids were extracted with 50% methanol at a 6:1 ratio. Extraction mixture was incubated at room temperature and then centrifugated at 4000 × *g* for 20 min. The supernatants were used for further chromatographic separation (using a Thermo Scientific UltiMate 3000 HPLC) and LC–MS detection (using a high-resolution tandem mass spectrometer Q-Exactive, Thermo Scientific). LC−MS raw data were converted into mzXML format and then processed by the XCMS with the R software. The mass difference threshold between observed and actual value was set as 10 ppm. Further analysis was conducted as previously described [24].

### Transmission electron microscopy (TEM)

TEM sample was prepared as previously described [37]. All images were obtained using a transmission electron microscope (JEM-1230, JEOL Ltd., Tokyo, Japan). The area, diameter, number, and average area of lipid droplets were calculated as previously described [38]. Images representing average level of corresponding group were chosen as representative ones. Ten representative images were used per group for analysis.

### Histological and morphological analysis

The heart tissue was fixed with 4% paraformaldehyde, dehydrated and embedded with paraffin. 5-μm-thick serial sections were generated. RTN3 immunohistochemical staining was performed according to standard procedure as previously described [39]. Anti-RTN3 (Abcam, # ab187764) was used in immunohistochemical staining. Left ventricular tissue was used for oil red O staining as previously described [11]. Image analysis and quantification were conducted using ImageJ software as previously described [11, 40]. Images representing average level of corresponding group were chosen as representative ones.

### Blood lipids measurement

Blood sample was stored at 4 °C for 8 h and then centrifugated at 3000 × *g* for 15 min to get serum. The serum was stored at −80 °C before analysis. The blood triglyceride (TG), cholesterol (CHO), high-density lipoprotein (HDL), and low-density lipoprotein (LDL) were measured using an automatic biochemical analyser (Chemray 800). Six mice were used per group for serum lipids analysis.

### Cell culture

Primary NRVCs were isolated from Sprague Dawley neonatal rat pups (0–3 days old) as previously described [37]. HEK293T cells were purchased from Merck(#VP001). Cells were cultured in the Dulbecco’s modified Eagle’s medium (DMEM) supplemented with 10% fetal bovine serum (FBS) and 1% penicillin/streptomycin. To investigate the effect of palmitate, cells were incubated with palmitate (500 μmol/L) for indicated time.

Adult ventricular cardiomyocytes were isolated by enzymatic dissociation as described previously [41]. The collected adult cardiomyocytes were then subjected to western blotting analysis and immunofluorescence assay.

### Down-regulation and upregulation of target genes

Adenovirus expressing rat RTN3 gene (Ad RTN3), adenovirus expressing rat RTN3 small hairpin RNA (Ad sh RTN3), adenovirus expressing rat RTN3 1–194aa (Ad R1), adenovirus expressing rat RTN3 65–236aa (Ad R2), adenovirus expressing rat RTN3 65–194aa (Ad R3), and empty adenovirus (Ad NC) were generated by Shanghai Hanbio Co, Ltd. The sequence of Ad sh-RTN3 was: top strand was TCGAGGCCAAGCTGTGCAGAAGTCAGAAGAATTCAAGAGATTCTTCTGACTTCTGCACAGCTTGGTTTTTTA and bottom strand was AGCTTAAAAAACCAAGCTGTGCAGAAGTCAGAAGAATCTCTTGAATTCTTCTGACTTCTGCACAGCTTGGCC. For adenovirus transfection, cells were incubated with DMEM containing adenovirus for 6-8 h (multiplicity of infection: 50). 48-72 h after transfection, cells were collected for further analysis.

Small interfering RNA of FABP5 (siFABP5), DGAT1(siDGAT1), DGAT2(siDGAT2) were generated by Genepharma, China. siC/EBPα was generated by TSINGKE, China. The sequences of siRNAs were listed as below: the sequence of siFABP5 was GGAGAGAAGUUUGAUGAAATT (sense) and UUUCAUCAAACUUCUCUCCTT (anti-sense), the sequence of siDGAT1 was CCUACCGAGAUCUCUAUUATT (sense) and UAAUAGAGAUCUCGGUAGGTT (anti-sense), the sequence of siDGAT2 was CCACCGAAGUUAGCAAGAATT (sense) and UUCUUGCUAACUUCGGUGGTT (anti-sense), the sequence of siC/EBPα was GGCCGCUGGUGAUCAAGCATT (sense) and UGCUUGAUCACCAGCGGCCTT (anti-sense). For small interfering RNA transfection, Lipofectamine™ RNAiMAX (Invitrogen, #13778075) was used following manufacturer’s instructions.

pEX-3-RTN3-3xflag, pEX-3-R1-3xflag, pEX-3-R2-3xflag, pEX-3-R3-3xflag, and pEX-3-FABP5-myc were generated by Genepharma, China. For plasmids transfection, Lipofectamine™ 2000 (Invitrogen, #11668019) was used following manufacturer’s instructions.

### Western blotting

Cells or ventricular tissues were lysed using RIPA Lysis Buffer (Beyotime, # P0013B) supplemented with 1% phenylmethanesulfonylfluoride fluoride (PMSF, Beyotime, #ST506) following manufacturer’s instructions. Protein concentration was determined using Pierce BCA Protein Assay Kit (Thermo Scientific, #23225). Equal protein was loaded onto SDS-PAGE, separated and transferred to nitrocellulose membranes. Then the membranes were blocked with 5–10% skim milk in 1×TBST and incubated with primary antibodies at 4 °C overnight. After primary antibodies incubation, the membranes were incubated with secondary antibodies at room temperature for 1.5–2 h. A chemiluminescent detection system (Image Lab, Bio-Rad, US) was used to visualize and analyze the final results. Antibodies used in this study include anti-RTN3 (Abcam, #ab187764), anti-DDDDK tag (Abcam, #ab205606), anti-Myc tag (Abcam, #ab206486), anti-FABP5 (Proteintech, #12348-1-AP), anti-C/EBPα (Proteintech, #18311-1-AP), anti-CD36 (Affinity, #DF13262), anti-β-Actin (Proteintech, #66009-1-Ig), HRP-conjugated Affinipure Goat Anti-Rabbit IgG(H + L) (Proteintech, #SA00001-2), HRP-conjugated Affinipure Goat Anti-Mouse IgG(H + L) (Proteintech, #SA00001-1), HRP-conjugated Goat Anti-Rabbit IgG HCS (Abbkine, #A25222), and HRP-conjugated Goat Anti-Mouse IgG HCS (Abbkine, #A25112).

### Fluorescent imaging of fatty acid (FA), and LD

NRVCs were seeded on glass-bottomed culture dishes (NEST Biotechnology, # 801002). For FAs visualization, BODIPY™ 558/568 C_12_(Invitrogen, # D3835) was used following manufacturer’s instructions. Briefly, cells were incubated with complete medium containing 1 μM BODIPY™ 558/568 C_12_ for 16 h. Before imaging, the medium was discarded and cells were wash with HBSS buffer for three time to remove the residual dye. For LD visualization, BODIPY™ 493/503 NHS Ester (Invitrogen, # D2191) was used following manufacturer’s instructions. Briefly, live cells or fixed cells were incubated with complete medium or PBS buffer containing 200 ng/ml BODIPY™ 493/503 NHS Ester for 1 min. Then the medium or PBS buffer was discarded and cells were wash with HBSS buffer for three time to remove the residual dye. All fluorescent images were obtained using a confocal laser-scanning microscope (Nikon A1 plus Confocal Microscope, Nikon, Japan). ImageJ colocalization finder was used to analyze the overlap coefficient between FFA and LD. ImageJ was used to calculate the area of LD.

### Immunofluorescence microscopy

Immunofluorescence was conducted as previously described [16]. Antibodies used in this study include anti-RTN3 (Santa Cruz, #sc-374599), anti-FABP5 (Proteintech, #12348-1-AP), anti-DGAT2 (Proteintech, #17100-1-AP), Cy3–conjugated Affinipure Goat Anti-Mouse IgG(H + L) (Proteintech, # SA00009-1), and DyLight 405-labeled Goat Anti-Rabbit IgG(H + L) (Beyotime, # A0605). ImageJ was used to calculate the area of isolated cardiomyocytes. Ten representative images were used per group for analysis.

### Co-immunoprecipitation (Co-IP)

NRVCs and ventricular tissues were used for Co-IP. Co-IP was conducted using Pierce Classic Magnetic IP/Co-IP Kit (Thermo Scientific, #88804) following manufacturer’s instructions. Antibodies used in this study include anti-RTN3 (Abcam, #ab187764), anti-FABP5 (Proteintech, #12348-1-AP) and normal rabbit IgG (Beyotime, #A7016).

### Liquid chromatography tandem mass spectrometry (LC-MS/MS) analysis for RTN3-interacting proteins

Protein samples collected from RTN3 immunoprecipitation was used for LC–MS/MS analysis. Protein samples collected from IgG immunoprecipitation was used as negative control. LC–MS/MS analysis was conducted as previously described [37]. Q Exactive HF-X mass spectrometer (Thermo Fisher) and EASY-nLC 1200 UHPLC system (Thermo Fisher) were used in LC-MS/MS analysis.

### Chromatin immunoprecipitation (ChIP)-quantitative polymerase chain reaction (qPCR) assay

ChIP was conducted using Simple ChIP Enzymatic Chromatin IP Kit (Cell Signaling Technology, #9003) following manufacturer’s instructions. Briefly, cells, treated with palmitate or not, were fixed with formaldehyde and then lysed. Chromatin was harvested and fragmented using enzymatic digestion. The fragmented chromatin was then subjected to immunoprecipitation using either anti-C/EBPα (Proteintech, #18311-1-AP) or IgG (Beyotime, #A7016). After immunoprecipitation, the protein-DNA cross-links were reversed and purified DNA was collected. The obtained DNA was subjected to agarose gel electrophoresis analysis and qPCR analysis. The qPCR assay was conducted using TB Green Premix Ex Taq II kit (Takara, #RR820A) and a PCR System (Bio-Rad, US). The sequences of specific primers to RTN3 promoter binding region were: forward: 5′-TCTTTCTCTGGCACTCAACGG-3′ and reverse: 5′- CAGATTGCCTTCTGGTTATTGCAAT-3′.

### Real-time (RT)-PCR

RNA of cells or ventricular tissues was extracted with RNAisoPlus (Takara, #9189Q) following manufacturer’s instructions. The reverse transcription of RNA was conducted using PrimeScriptTM RT Reagent Kit (Takara, #RR047A). RT–PCR was conducted using TB Green Premix Ex Taq II kit (Takara, #RR820A) and CFX Real-Time PCR System (Bio-Rad, US). The sequences of specific primers to rat RTN3 were: forward: 5′- CTTCATGTGGCTGATGACCTATG-3′ and reverse: 5′-TAGACAATTGGGACGCTGAAGA-3′.

### Dual-luciferase reporter assay

Plasmids, including pcDNA3.1-negative control (pcDNA3.1-NC), pcDNA3.1- C/EBPα, pGL-Basic, and pGL-RTN3 promoter region, were constructed by HanBio technology (Shanghai, China). Sequences of all plasmids were verified. Dual-Luciferase® Reporter Assay System (Promega, # E1910) was used to assess the luciferase activity. HEK-293T cells were transfected with plasmids as displayed in results and then cells were collected following manufacturer’s instructions. The luciferase activity was measured with GloMax96 plate reader (Biotek, USA).

To identify the specific region targeted by C/EBPα, pGL-RTN3-0 (−2000 to 0 bp), pGL-RTN3-1 (−1450 to 0 bp), pGL-RTN3-2 (−750 to 0 bp), and pGL-RTN3-3 (−450 to 0 bp) were constructed by HanBio technology (Shanghai, China). HEK-293T cells were transfected with above plasmids and pcDNA3.1- C/EBPα and then were collected for luciferase activity detection. Then plasmid expressing RTN3 promotor region with “GTGACTGCATTG” deleted was constructed and transfected.

### Study population

The diagnostic criteria of obesity were according to World Health Organization (WHO). From January 2020 to June 2022, obese patients (body mass index, BMI, >30 kg/m^2^) and people with normal weight (18.5 kg/m^2^ <BMI ≤ 24.9 kg/m^2^, defined as normal weight, NW) were consecutively recruited at the Department of Cariology, Tangdu Hospital, Shannxi, China. The following patients were excluded: ① signs of severe cardiac dysfunction (i.e., left ventricular ejection fraction <40%, clinical signs of heart failure, or pro-brain natriuretic peptide level >100 ng/L); ② stenosis >50% of any coronary artery; ③ women in pregnancy or lactation. Clinical data, echocardiographic data and laboratory data were collected at the time of admission. Right atrial tissues were obtained during cannulation before cardiopulmonary bypass. Once removed, tissues were immediately frozen in liquid nitrogen or fixed in 4% paraformaldehyde for further analysis. The study protocol was approved by Local Ethics Committee (IEC of Institution for National Drug Clinical Trails, Tangdu Hospital, Air Force Medical University, No.202103-12). All participants were fully aware of the goal and possible risk of this study and provided written informed consent.

### Statistical analysis

All values were displayed as the mean ± standard error of mean (SEM). For continuous data, unpaired two-tailed Student’s *t* test was used in two groups analysis, and one-way analysis of variance (ANOVA) was used with a Bonferroni post hoc test in multiple group analysis. For categorical data, the *χ*^2^ test was performed when the total number was above 40, and the Fisher’s exact test was performed when the total number was under 40. Above analysis was conducted with GraphPad Prism 6.0 software (GraphPad Software, La Jolla, USA). A value of probability (P) < 0.05 was considered statistically significant.

### Supplementary information

Supplementary information

Supplementary Video 1

Supplementary Video 2

Supplementary Video 3

Original Data File

reproducibility checklist

## Data Availability

All source data generated during this study are provided with this article.
