# Supplementary material for: Lipid overload-induced RTN3 activation leads to cardiac dysfunction by promoting lipid droplet biogenesis
Source: Cell Death Differ. 2023 Nov 28;31(3):292–308. doi: 10.1038/s41418-023-01241-x (PMC10923887; doi:10.1038/s41418-023-01241-x)
Supplement: Supplementary file 1 — Supplementary information [file 41418_2023_1241_MOESM1_ESM.docx]

**Supplementary figures and figure legends**


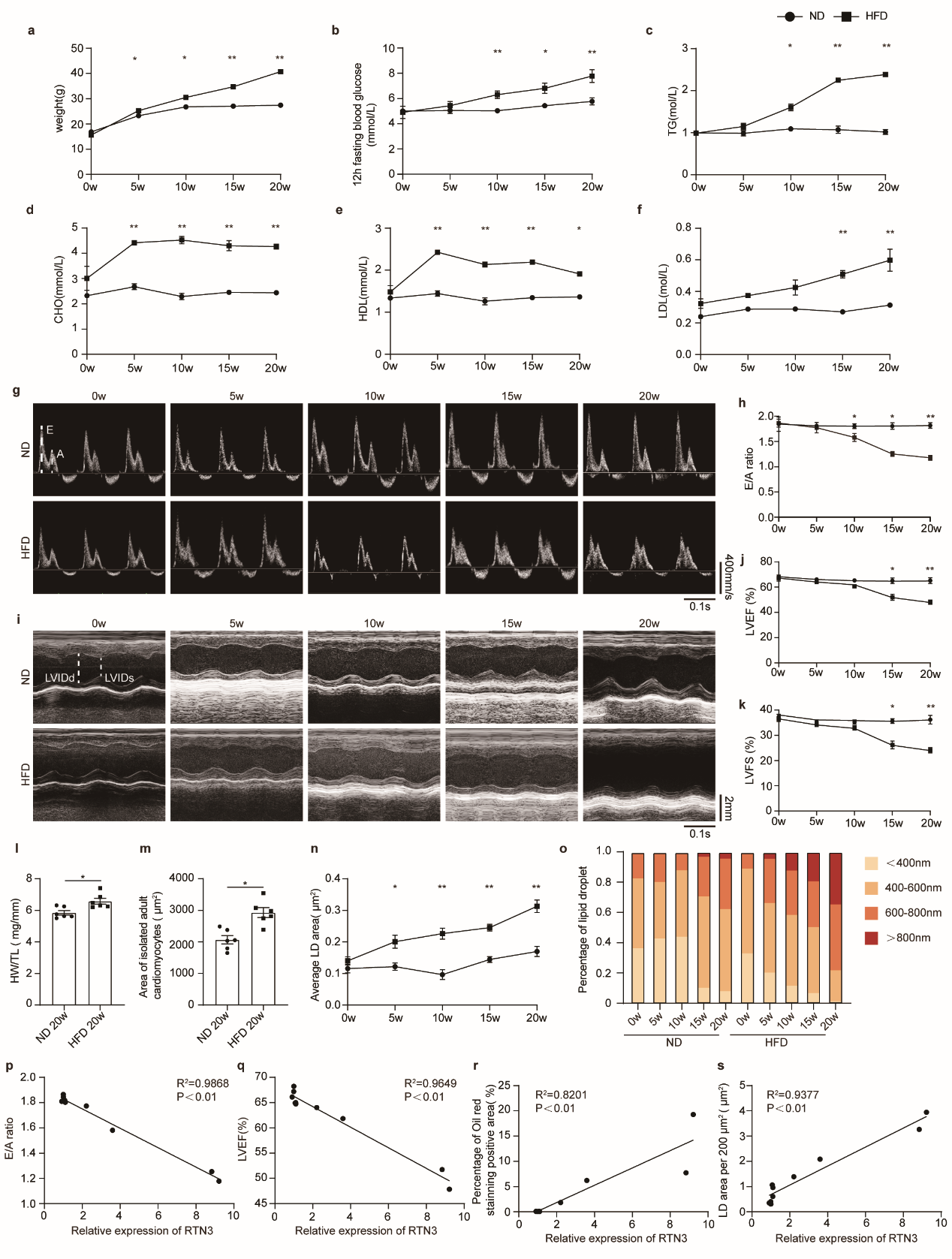


**Supplementary Fig.1: WT mice were fed with ND or HFD for 20 weeks.** **a–f** Quantitative analysis of body weight (a), 12h blood serum glucose (b), TG (c), CHO (d), HDL (e), and LDL (f) of WT mice every 5 weeks throughout the 20-week ND or HFD (n=6 each group); **g–k** echocardiographic assessments were performed on ND- and HFD-fed WT mice. (n=6 mice each group); (g) Representative doppler echocardiography images of wild type mice every 5 weeks throughout the 20-week ND or HFD; (h) Quantitative analysis of E/A ratio; (i) Representative M-mode echocardiography images of wild type mice every 5 weeks throughout the 20-week ND or HFD; (j&k) Quantitative analysis of LVEF and LVFS; **l** Quantitative analysis of HW/TL of ND-fed and HFD-fed WT mice (n=6 mice each group); **m** Quantitative analysis of area of isolated adult cardiomyocytes of ND-fed and HFD-fed WT mice (n=6 mice each group); **n** Quantitative analysis of average LD area (n=10 images each group); **o** Quantitative analysis of LD diameter calculated with TEM images (n=10 images each group); **p–s** Correlations between the protein level of RTN3 and E/A ratio (p), LVEF (q), positive area of oil red staining (r), and LD area per 200 μm^2^ (s). Data are expressed as Mean ± SEM. Differences are significant for *P<0.05, **P<0.01.


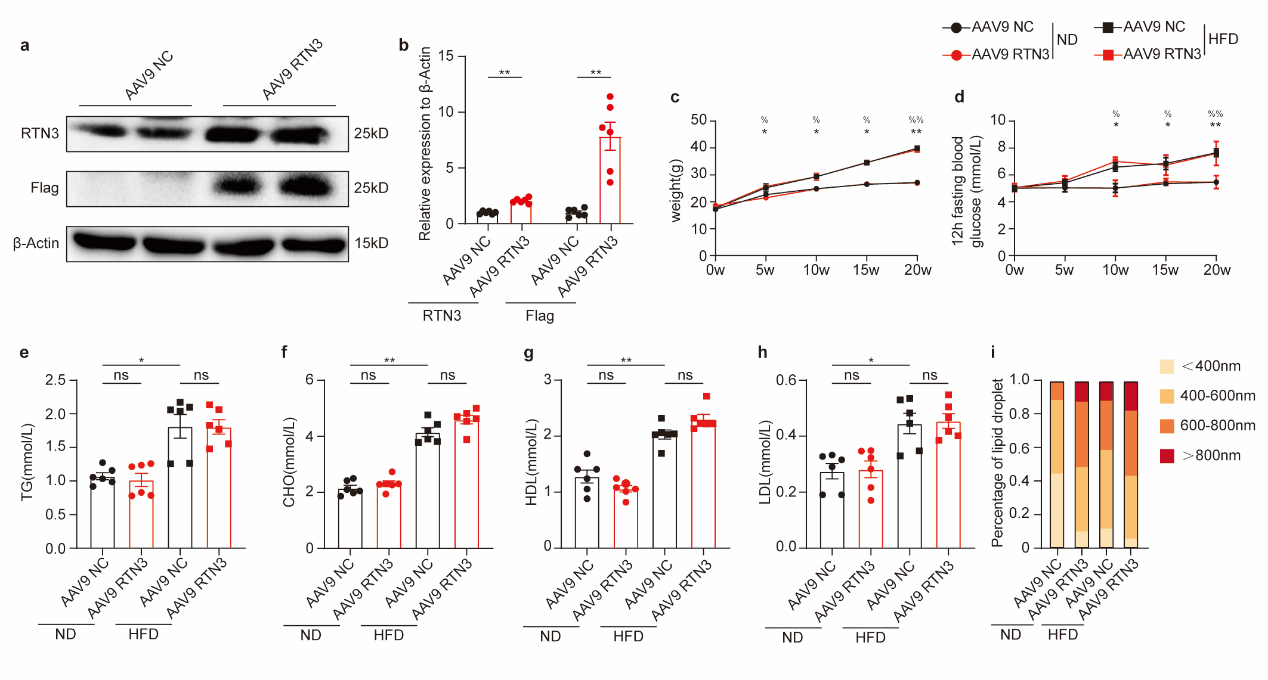


**Supplementary Fig.2: Cardiomyocyte-specific overexpression of RTN3 did not alter the systematic metabolism. a and b** Representative western blotting images (a) and quantitative analysis (b) of RTN3 and flag protein level of the heart (n=6 mice each group); **c and d** Quantitative analysis of body weight (c) and 12-h fasting blood glucose (d) of AAV9 NC and AAV9 RTN3 mice every 5 weeks throughout the 20-week ND or HFD (n=6 mice each group); **e–h** Quantitative analysis of TG (e), CHO (f), HDL (g), and LDL (h) of AAV9 NC and AAV9 RTN3 mice after 10-week ND or HFD (n=6 mice each group); **i** Quantitative analysis of LD diameter calculated with TEM images (n=10 images each group). Data are expressed as Mean ± SEM. Differences are significant for *P<0.05, **P<0.01.


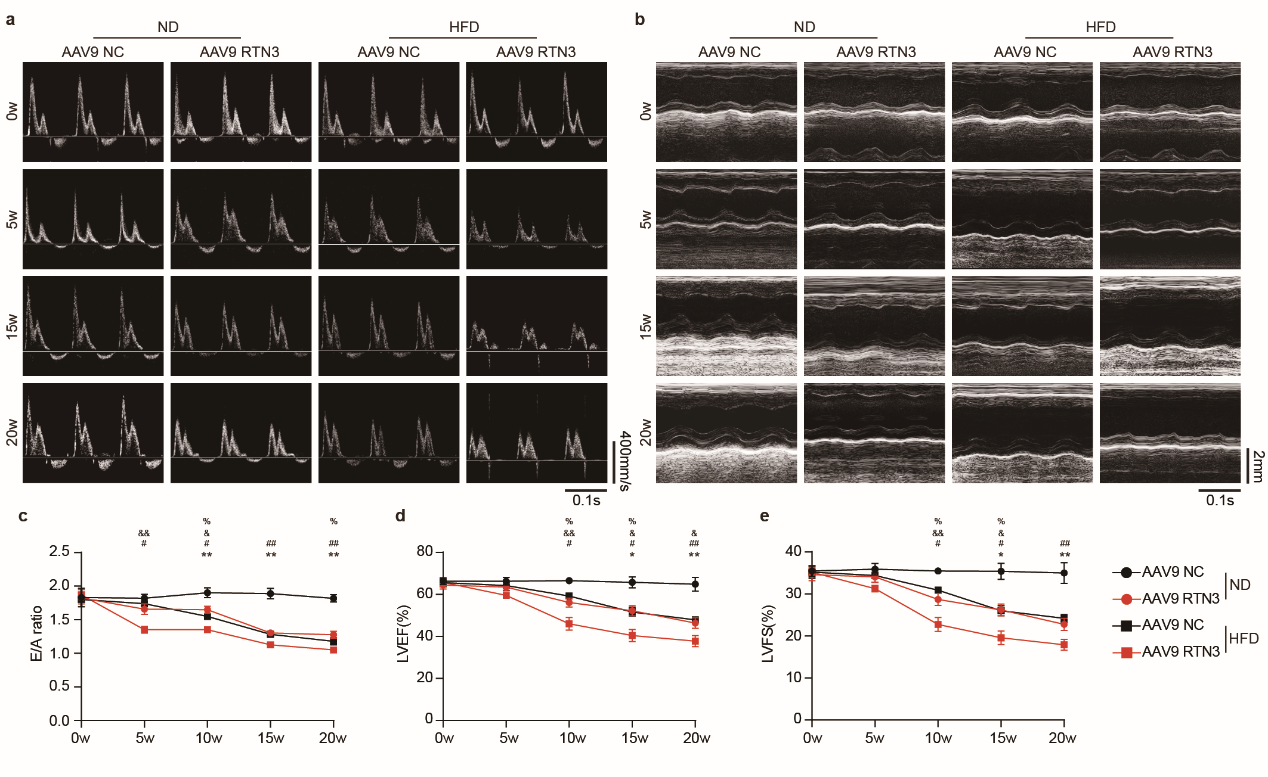


**Supplementary Fig.3: Cardiac systolic and diastolic function were monitored by echocardiography every 5weeks throughout the 20-week ND or HFD.** **a** Representative doppler echocardiography images of AAV9 NC and AAV9 RTN3 mice every 5 weeks throughout the 20-week ND or HFD; **b** Representative M-mode echocardiography images of AAV9 NC and AAV9 RTN3 mice every 5 weeks throughout the 20-week ND or HFD; **c–e** Quantitative analysis of E/A ratio (c), LVEF (d), and LVFS (e) (n=6 mice each group). Data are expressed as Mean ± SEM. * P<0.05, AAV9 NC ND vs. AAV9 NC HFD; ** P<0.01, AAV9 NC ND vs. AAV9 NC HFD; # P<0.05, AAV9 NC ND vs. AAV9 RTN3 ND; ## P<0.01, AAV9 NC ND vs. AAV9 RTN3 ND; & P<0.05, AAV9 NC HFD vs. AAV9 RTN3 HFD; && P<0.01, AAV9 NC HFD vs. AAV9 RTN3 HFD; % P<0.05, AAV9 RTN3 ND vs. AAV9 RTN3 HFD; %% P<0.01, AAV9 RTN3 ND vs. AAV9 RTN3 HFD.


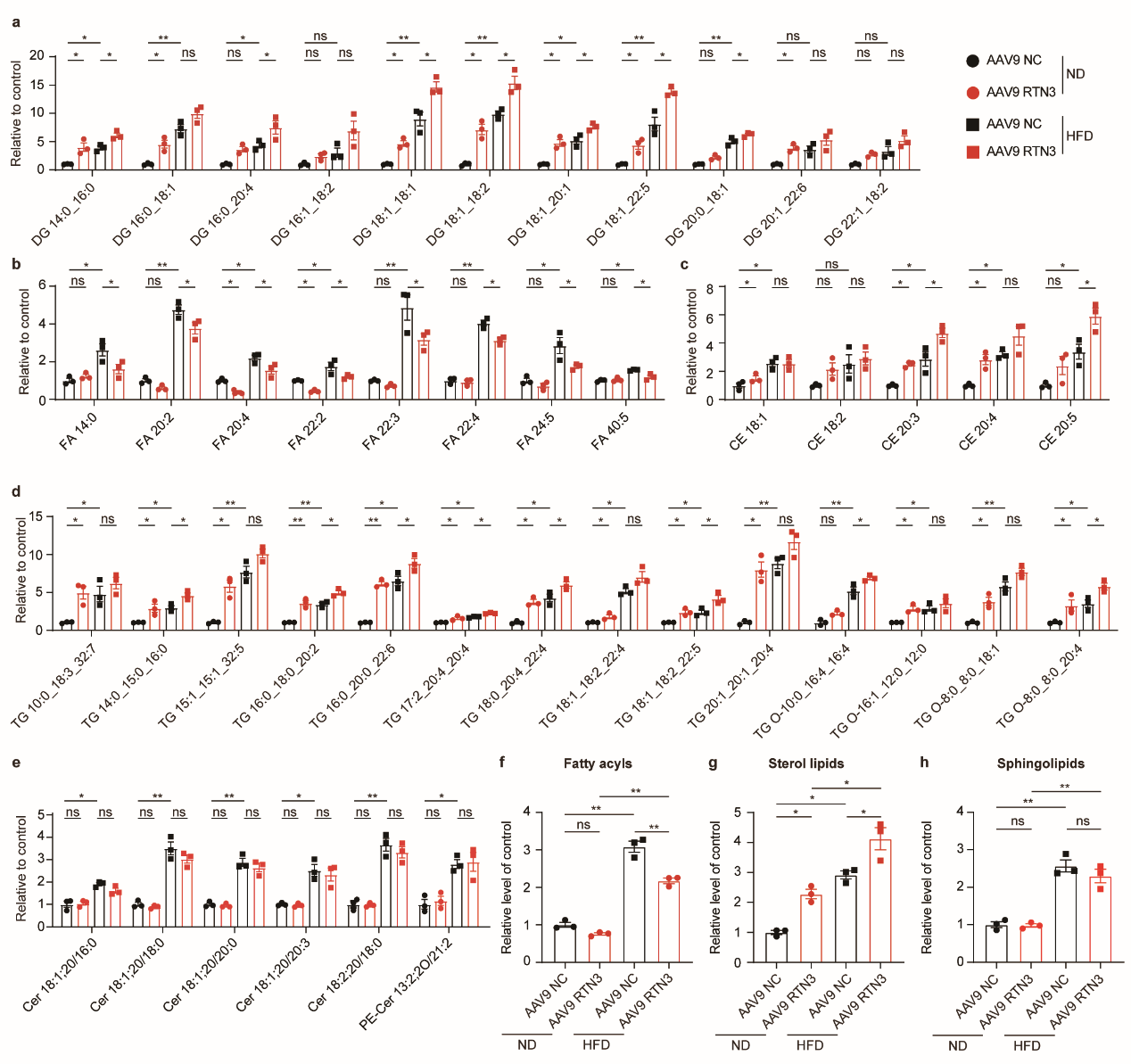


**Supplementary Fig.4: Cardiomyocyte-specific overexpression of RTN3 aggravated intramyocardial lipid accumulation. a–e** Quantitative analysis of significantly altered diacylglycerol (a), fatty acyls (b), sterol lipids (c), triacylglycerol (d), and sphingolipids (e) (n=3 mice each group); **f–h** Quantitative analysis of total fatty acyls (f), sterol lipids (g) and sphingolipids (h) (n=3 mice each group). Data are expressed as Mean ± SEM. Differences are significant for *P<0.05, **P<0.01.


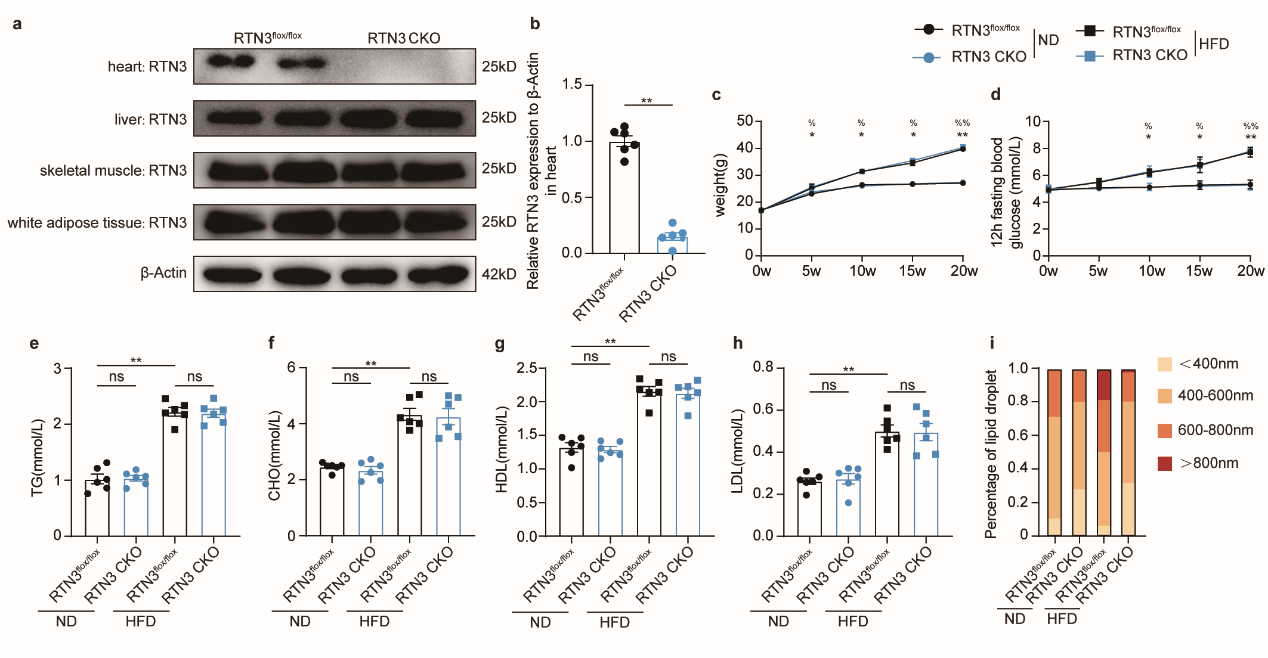


**Supplementary Fig.5: Cardiomyocyte-specific knockout of RTN3 did not alter the systematic metabolism. a and b** Representative western blotting images (a) and quantitative analysis (b) of RTN3 protein level (n=6 mice each group); **c and d** Quantitative analysis of body weight (c) and 12-h fasting blood glucose (d) of RTN3 ^flox/flox^ and RTN3 CKO mice every 5 weeks throughout the 20-week ND or HFD (n=6 mice each group); **e–h** Quantitative analysis of TG (e), CHO (f), HDL (g), and LDL (h) of RTN3 ^flox/flox^ and RTN3 CKO mice after the 15-week ND or HFD (n=6 mice each group); **i** Quantitative analysis of LD diameter calculated with TEM images (n=10 images each group). Data are expressed as Mean ± SEM. Differences are significant for *P<0.05, **P<0.01.


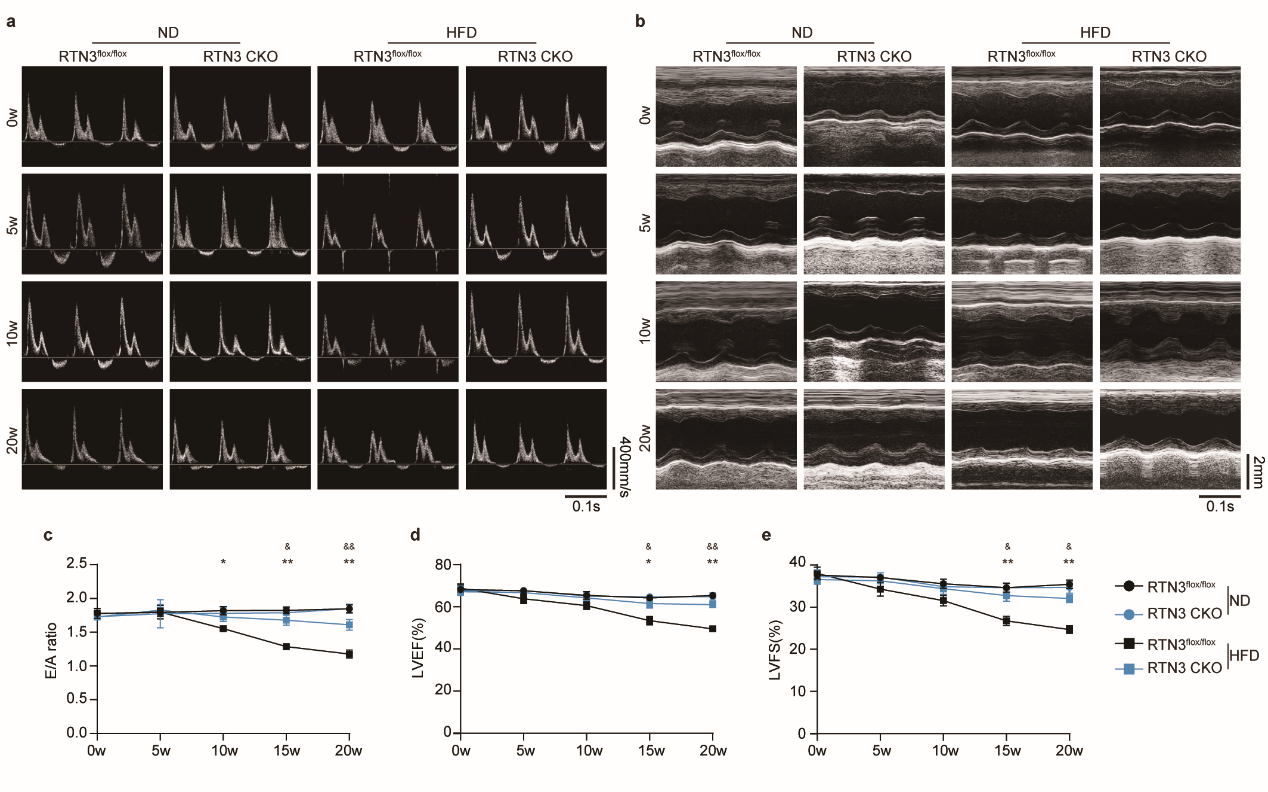


**Supplementary Fig.6: Cardiac systolic and diastolic function were monitored by echocardiography every 5weeks throughout the 20-week ND or HFD.** **a** Representative doppler images of RTN3 ^flox/flox^ and RTN3 CKO mice every 5 weeks throughout the 20-week ND or HFD; **b** Representative M-mode echocardiography images of RTN3 ^flox/flox^ and RTN3 CKO mice every 5 weeks throughout the 20-week ND or HFD; **c–e** Quantitative analysis of E/A ratio (c), LVEF (d), and LVFS (e) (n=6 mice each group). Data are expressed as Mean ± SEM. * P<0.05, RTN3 ^flox/flox^ ND vs. RTN3 ^flox/flox^ HFD; ** P<0.01, RTN3 ^flox/flox^ ND vs. RTN3 ^flox/flox^ HFD; & P<0.05, RTN3 ^flox/flox^ HFD vs. RTN3 CKO HFD; && P<0.01, RTN3 ^flox/flox^ HFD vs. RTN3 CKO HFD.


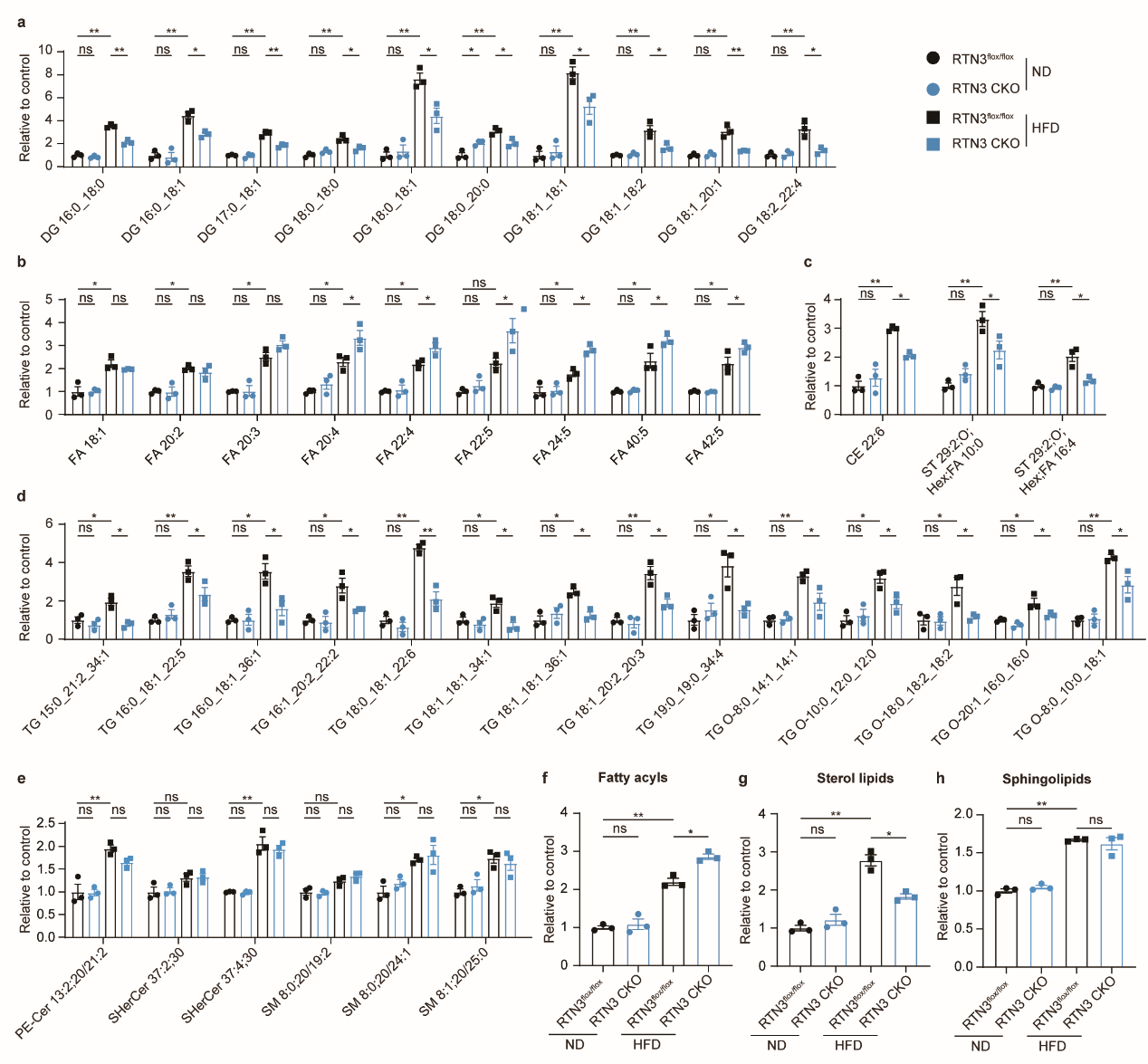


**Supplementary Fig.7: Cardiomyocyte-specific knockout of RTN3 alleviated HFD induced intramyocardial lipid accumulation. a–e** Quantitative analysis of significantly altered diacylglycerol (a), fatty acyls (b), sterol lipids (c), triacylglycerol (d), and sphingolipids (e) (n=3 mice each group); **f–h** Quantitative analysis of total fatty acyls (f), sterol lipids (g) and sphingolipids (h) (n=3 mice each group). Data are expressed as Mean ± SEM. Differences are significant for *P<0.05, **P<0.01.


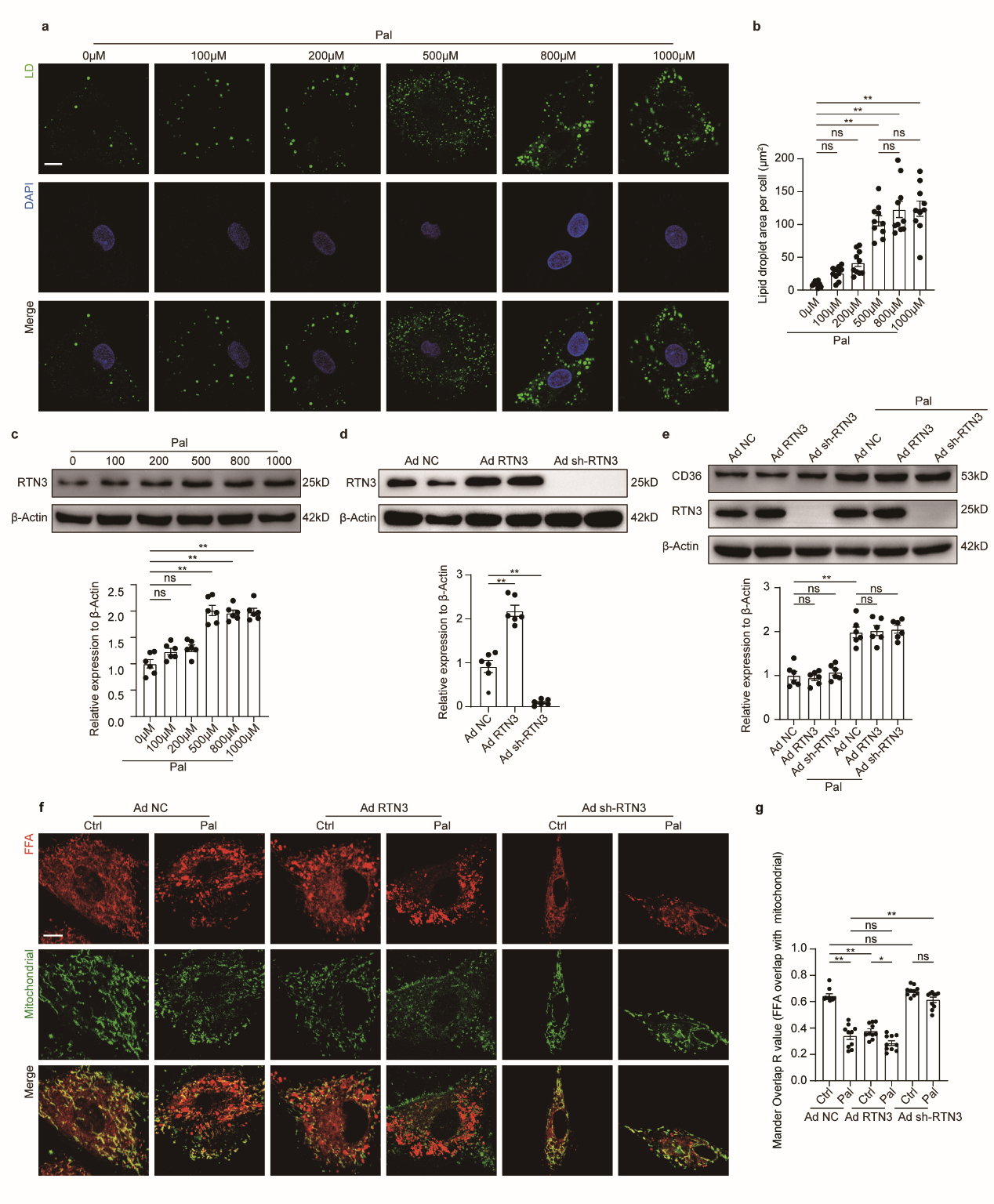


**Supplementary Fig.8: RTN3 affected the fate of intracellular FA. a and b** Representative fluorescence images indicating LDs labeled with Bodipy 493/503 and nucleus labeled with DAPI (a) and quantitative analysis of LDs area per cell (b) (n=10 images each group), scale bar=10μm; **c** Representative western blotting images and quantitative analysis of RTN3 protein expression with palmitate treatment (n=6 wells each group); **d** Representative western blotting images and quantitative analysis of RTN3 protein expression with adenovirus treatment (n=6 wells each group); **e** Representative western blotting images and quantitative analysis of CD36 protein expression (n=6 wells each group); **f and g** Representative fluorescence images indicating FFA labeled with Bodipy 558/568 C12 and mitochondrial labeled with Mito Tracker (f) and quantitative analysis of localization of FFA with mitochondrial quantified with Mandar overlap R value analysis (g) (n=10 images each group), scale bar=10μm. Data are expressed as Mean ± SEM. Differences are significant for *P<0.05, **P<0.01.


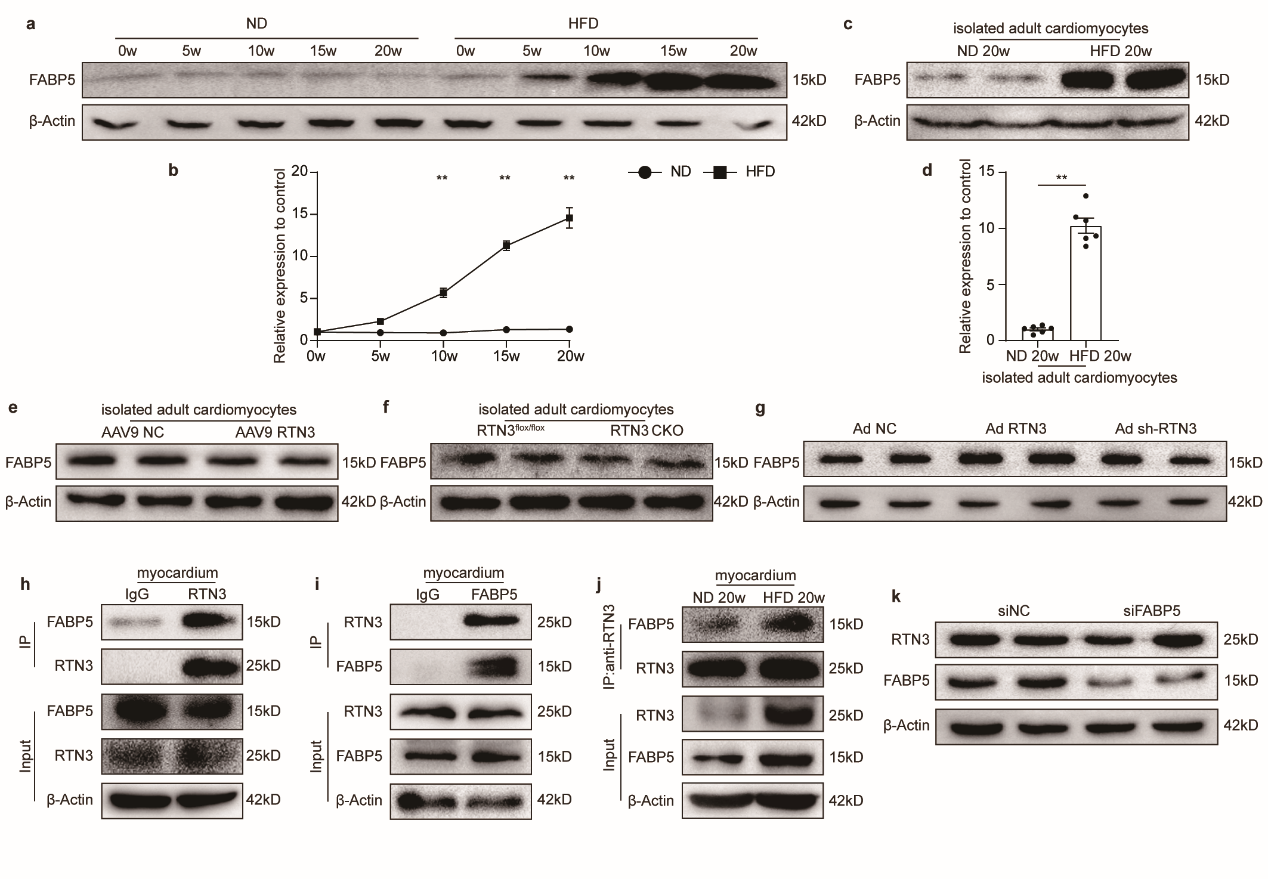


**Supplementary Fig.9: RTN3 directly interacted with FABP5 in vivo.** **a and b** Representative western blotting images (a) and quantitative analysis (b) of FABP5 protein expression in the hearts (n=6 mice each group); **c and d** Representative western blotting images (c) and quantitative analysis (d) of FABP5 protein expression in the isolated adult cardiomyocytes (n=6 mice each group); **e** Representative western blotting images of FABP5 protein expression in the isolated adult cardiomyocytes of RTN3 overexpression mice; **f** Representative western blotting images of FABP5 protein expression in the isolated adult cardiomyocytes of RTN3 knockout mice; **g** Representative western blotting images of FABP5 protein expression with adenovirus treatment; **h and i** Representative western blotting images of IP assay using anti-RTN3 (h) or anti-FABP5 (i) to determine the interaction of RTN3 and FABP5 in myocardium of mice; **j** Representative images of IP assay using anti-RTN3, the myocardium of mice was used to perform the IP assay; **k** Representative western blotting images of RTN3 protein expression with siFABP5 treatment. Data are expressed as Mean ± SEM. Differences are significant for **P<0.01.


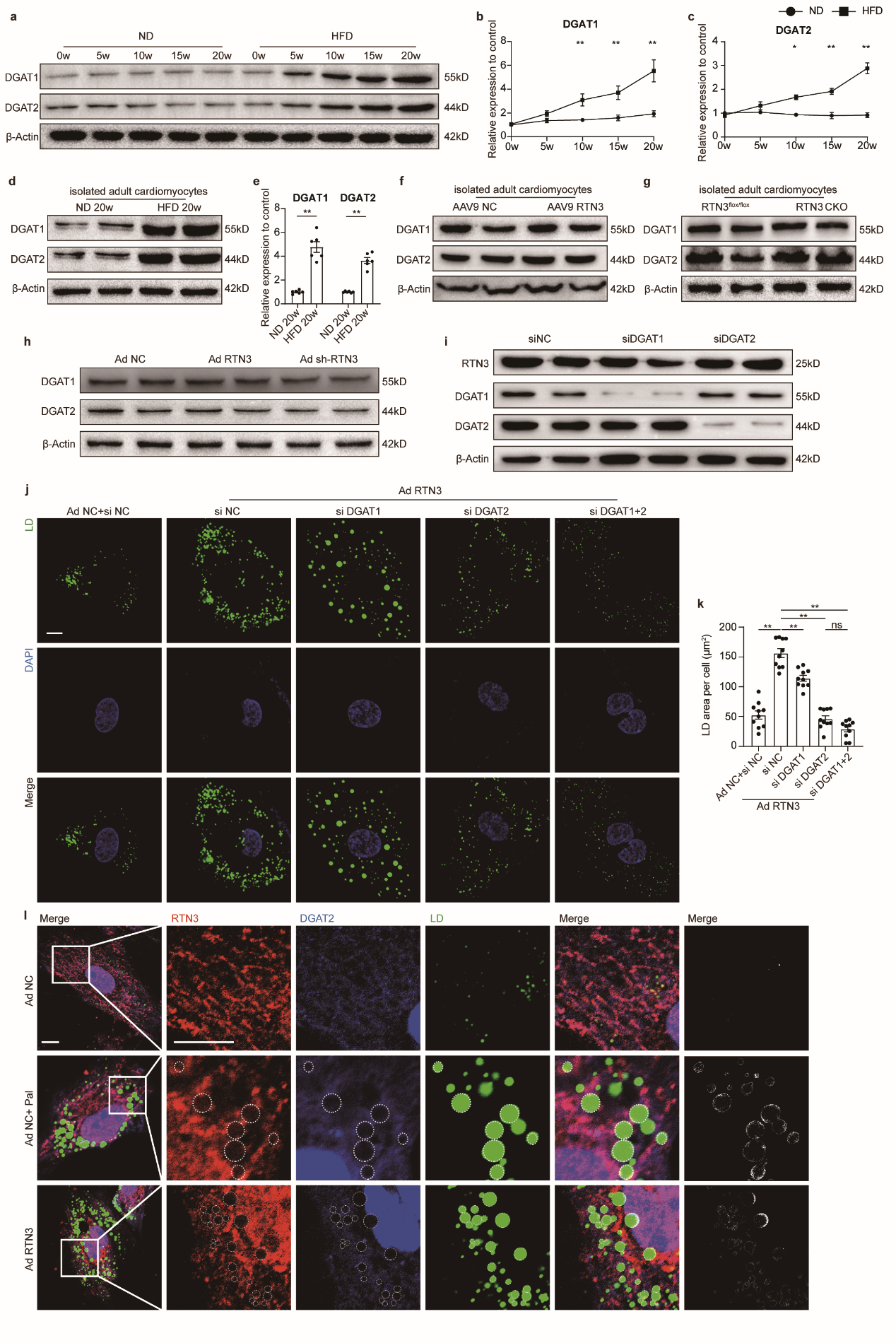


**Supplementary Fig.10: The RTN3-mediated LD biogenesis was dependent on DGAT2.** **a–c** Representative western blotting images (a) and quantitative analysis (b and c) of DGAT1 and DGAT2 protein expression in the hearts (n=6 mice each group); **d and e** Representative western blotting images (d) and quantitative analysis (e) of DGAT1 and DGAT2 protein expression in the isolated adult cardiomyocytes (n=6 mice each group); **f** Representative western blotting images of DGAT1 and DGAT2 protein expression in the isolated adult cardiomyocytes of RTN3 overexpression mice; **g** Representative western blotting images of DGAT1 and DGAT2 protein expression in the isolated adult cardiomyocytes of RTN3 knockout mice; **h** Representative western blotting images of DGAT1 and DGAT2 protein expression with adenovirus treatment; **i** Representative western blotting images of RTN3 protein expression with siDGAT1 or siDGAT2 treatment; **j and k** Representative fluorescence images indicating LDs labeled with Bodipy 493/503 and nucleus labeled with DAPI (j) and quantitative analysis of LDs area per cell (k) (n=10 each group), scale bar=10μm; **l** Representative immunofluorescence images indicating RTN3, DGAT2, and LD, scale bar=10μm. Data are expressed as Mean ± SEM. Differences are significant for **P<0.01.

**Supplementary Tables**

|  | E/A | LVEF (%) | LVFS (%) |
| --- | --- | --- | --- |
| ND |  |  |  |
| ND 0w | 1.85±0.14 | 68.24±1.12 | 37.33±0.93 |
| ND 5w | 1.81±0.07 | 66.12±1.07 | 35.36±0.81 |
| ND 10w | 1.80±0.05 | 65.02±1.09 | 35.11±0.83 |
| ND 15w | 1.81±0.06 | 64.74±2.19 | 34.78±1.66 |
| ND 20w | 1.82±0.05 | 64.92±2.35 | 35.30±1.71 |
| HFD |  |  |  |
| HFD 0w | 1.86±0.08 | 67.19±2.36 | 36.52±1.81 |
| HFD 5w | 1.77±0.11 | 64.03±0.85 | 34.10±0.61 |
| HFD 10w | 1.58±0.08* | 61.84±1.26 | 32.74±0.90 |
| HFD 15w | 1.25±0.05* | 51.73±2.49* | 26.05±1.54* |
| HFD 20w | 1.17±0.05** | 47.85±1.70** | 23.96±1.05** |

**Supplementary Table 1. Echocardiographic indices of LV systolic and diastolic function in** **wild type mice every 5 weeks throughout the 20-week ND or HFD (n=6 each group)**. All data were expressed as mean±SEM. ND, normal diet; HFD, high fat diet; LVEF, left ventricular ejection fraction; LVFS, left ventricular fractional shortening. Differences are significant for *P<0.05 ND vs. HFD, **P<0.01 ND vs. HFD.

|  | E/A | LVEF (%) | LVFS (%) |
| --- | --- | --- | --- |
| AAV9 NC, ND |  |  |  |
| AAV9 NC, ND, 0w | 1.83±0.14 | 66.3±1.6 | 35.48±1.27 |
| AAV9 NC, ND, 5w | 1.82±0.06 | 66.3±1.8 | 35.91±1.38 |
| AAV9 NC, ND, 10w | 1.90±0.07 | 66.6±0.9 | 35.48±0.46 |
| AAV9 NC, ND, 15w | 1.89±0.08 | 65.8±2.6 | 35.40±1.91 |
| AAV9 NC, ND, 20w | 1.82±0.06 | 64.9±3.3 | 35.00±2.49 |
| AAV9 RTN3, ND |  |  |  |
| AAV9 RTN3, ND, 0w | 1.82±0.09 | 64.50±1.93 | 34.62±1.43 |
| AAV9 RTN3, ND, 5w | 1.66±0.08^#^ | 63.49±1.77 | 34.09±1.25 |
| AAV9 RTN3, ND, 10w | 1.65±0.05^#^ | 56.14±2.16^#^ | 28.71±1.45^#^ |
| AAV9 RTN3, ND, 15w | 1.30±0.03^##^ | 52.21±2.42^#^ | 26.23±1.43^#^ |
| AAV9 RTN3, ND, 20w | 1.28±0.05^##^ | 46.34±2.58^##^ | 22.79±1.45^##^ |
| AAV9 NC, HFD |  |  |  |
| AAV9 NC, HFD, 0w | 1.80±0.05 | 65.65±2.01 | 35.22±1.47 |
| AAV9 NC, HFD, 5w | 1.74±0.04 | 64.20±2.37 | 34.43±1.64 |
| AAV9 NC, HFD, 10w | 1.55±0.02** | 59.25±0.89 | 30.93±0.63 |
| AAV9 NC, HFD, 15w | 1.28±0.05** | 51.61±2.03* | 26.01±1.21* |
| AAV9 NC, HFD, 20w | 1.18±0.05** | 47.91±1.41** | 24.22±0.95** |
| AAV9 RTN3, HFD |  |  |  |
| AAV9 RTN3, HFD, 0w | 1.87±0.08 | 65.76±1.61 | 35.18±1.28 |
| AAV9 RTN3, HFD, 5w | 1.35±0.05^&&^ | 59.57±1.13 | 31.29±0.82 |
| AAV9 RTN3, HFD, 10w | 1.35±0.03^& %^ | 46.05±3.01^&& %^ | 22.76±1.68^&& %^ |
| AAV9 RTN3, HFD, 15w | 1.13±0.04 | 40.45±2.90^& %^ | 19.59±1.60^& %^ |
| AAV9 RTN3, HFD, 20w | 1.05±0.04^%^ | 37.79±2.60^&^ | 17.91±1.31 |

**Supplementary Table 2. Echocardiographic indices of LV systolic and diastolic function in AAV9 NC and AAV9 RTN3 mice every 5 weeks throughout the 20-week ND or HFD (n=6 each group).** All data were expressed as mean±SEM. ND, normal diet; HFD, high fat diet; AAV9, adeno-associated virus 9; LVEF, left ventricular ejection fraction; LVFS, left ventricular fractional shortening. Differences are significant for * P<0.05, AAV9 NC ND vs. AAV9 NC HFD; ** P<0.01, AAV9 NC ND vs. AAV9 NC HFD; ^#^ P<0.05, AAV9 NC ND vs. AAV9 RTN3 ND; ^##^ P<0.01, AAV9 NC ND vs. AAV9 RTN3 ND; ^&^ P<0.05, AAV9 NC HFD vs. AAV9 RTN3 HFD; ^&&^ P<0.01, AAV9 NC HFD vs. AAV9 RTN3 HFD; ^%^ P<0.05, AAV9 RTN3 ND vs. AAV9 RTN3 HFD; ^%%^ P<0.01, AAV9 RTN3 ND vs. AAV9 RTN3 HFD.

|  | E’/A’ |
| --- | --- |
| AAV9 NC, ND, 10w | 0.96±0.04 |
| AAV9 RTN3, ND, 10w | 0.61±0.04^##^ |
| AAV9 NC, HFD, 10w | 0.63±0.04** |
| AAV9 RTN3, HFD, 10w | 0.44±0.03^& %^ |

**Supplementary Table 3. Tissue doppler-derived echocardiographic indices of LV systolic and diastolic function in AAV9 NC and AAV9 RTN3 mice after 10-week ND or HFD (n=6 each group).** All data were expressed as mean±SEM. ND, normal diet; HFD, high fat diet; AAV9, adeno-associated virus 9. Differences are significant for ** P<0.01, AAV9 NC ND vs. AAV9 NC HFD; ^##^ P<0.01, AAV9 NC ND vs. AAV9 RTN3 ND; ^&^ P<0.05, AAV9 NC HFD vs. AAV9 RTN3 HFD; ^%^ P<0.05, AAV9 RTN3 ND vs. AAV9 RTN3 HFD.

|  | E/A | LVEF (%) | LVFS (%) |
| --- | --- | --- | --- |
| RTN3^flox/flox^, ND |  |  |  |
| RTN3^flox/flox^, ND, 0w | 1.78±0.08 | 68.24±1.12 | 37.62±0.86 |
| RTN3^flox/flox^, ND, 5w | 1.79±0.06 | 67.62±1.01 | 37.01±0.66 |
| RTN3^flox/flox^, ND, 10w | 1.82±0.06 | 65.40±1.80 | 35.56±1.07 |
| RTN3^flox/flox^, ND, 15w | 1.82±0.05 | 64.21±1.43 | 34.61±1.04 |
| RTN3^flox/flox^, ND, 20w | 1.85±0.06 | 65.37±1.30 | 35.43±1.02 |
| RTN3 CKO, ND |  |  |  |
| RTN3 CKO, ND, 0w | 1.73±0.03 | 67.56±2.16 | 37.23±1.67 |
| RTN3 CKO, ND, 5w | 1.77±0.21 | 67.76±1.19 | 37.15±1.01 |
| RTN3 CKO, ND, 10w | 1.78±0.09 | 65.13±1.77 | 34.85±1.24 |
| RTN3 CKO, ND, 15w | 1.79±0.08 | 64.79±1.52 | 34.63±1.16 |
| RTN3 CKO, ND, 20w | 1.85±0.07 | 64.83±1.73 | 34.68±1.37 |
| RTN3^flox/flox^, HFD |  |  |  |
| RTN3^flox/flox^, HFD, 0w | 1.78±0.07 | 68.86±2.05 | 37.97±1.55 |
| RTN3^flox/flox^, HFD, 5w | 1.80±0.10 | 63.85±2.09 | 34.27±1.69 |
| RTN3^flox/flox^, HFD, 10w | 1.55±0.03* | 60.54±1.86 | 31.56±1.25 |
| RTN3^flox/flox^, HFD, 15w | 1.29±0.05** | 53.43±1.95* | 26.76±1.10** |
| RTN3^flox/flox^, HFD, 20w | 1.18±0.06** | 49.57±1.49** | 24.68±0.95** |
| RTN3 CKO, HFD |  |  |  |
| RTN3 CKO, HFD, 0w | 1.73±0.05 | 67.14±1.50 | 36.55±1.09 |
| RTN3 CKO, HFD, 5w | 1.83±0.09 | 66.79±1.52 | 36.25±1.28 |
| RTN3 CKO, HFD, 10w | 1.73±0.07 | 64.24±1.70 | 34.38±1.35 |
| RTN3 CKO, HFD, 15w | 1.68±0.07^&^ | 61.56±1.93^&^ | 32.74±1.33^&^ |
| RTN3 CKO, HFD, 20w | 1.61±0.08^&&^ | 61.04±1.36^&&^ | 32.04±0.93^&^ |

**Supplementary Table 4. Echocardiographic indices of LV systolic and diastolic function in RTN3^flox/flox^ and RTN3 CKO mice every 5 weeks throughout the 20-week ND or HFD (n=6 each group).** All data were expressed as mean±SEM. ND, normal diet; HFD, high fat diet; CKO, conditional cardiomyocyte-specific knockout; LVEF, left ventricular ejection fraction; LVFS, left ventricular fractional shortening. Differences are significant for * P<0.05, RTN3 ^flox/flox^ ND vs. RTN3 ^flox/flox^ HFD; ** P<0.01, RTN3 ^flox/flox^ ND vs. RTN3 ^flox/flox^ HFD; ^&^ P<0.05, RTN3 ^flox/flox^ HFD vs. RTN3 CKO HFD; ^&&^ P<0.01, RTN3 ^flox/flox^ HFD vs. RTN3 CKO HFD.

|  | E’/A’ |
| --- | --- |
| RTN3^flox/flox^, ND, 15w | 0.95±0.05 |
| RTN3 CKO, ND, 15w | 0.87±0.02 |
| RTN3^flox/flox^, HFD, 15w | 0.62±0.03** |
| RTN3 CKO, HFD, 15w | 0.75±0.02^&^ |

**Supplementary Table 5. Tissue doppler-derived echocardiographic indices of LV systolic and diastolic function in RTN3^flox/flox^ and RTN3 CKO mice after 15-week ND or HFD (n=6 each group).** All data were expressed as mean±SEM. ND, normal diet; HFD, high fat diet; CKO, conditional cardiomyocyte-specific knockout. Differences are significant for ** P<0.01, RTN3 ^flox/flox^ ND vs. RTN3 ^flox/flox^ HFD; ^&^ P<0.05, RTN3 ^flox/flox^ HFD vs. RTN3 CKO HFD.

| Protein name | Molecular mass(kDa) | Abundance | Abundance rank |
| --- | --- | --- | --- |
| Fatty acid binding protein 5 | 15.16 | 5.4×10^7^ | 1 |
| Histone H1.5 | 22.58 | 4.3×10^7^ | 2 |
| 60S ribosomal protein L23a | 17.70 | 3.6×10^7^ | 3 |
| Lysosomal associated membrane protein 1 | 43.87 | 2.5×10^7^ | 4 |
| Ribosomal protein L7 | 29.23 | 2.3×10^7^ | 5 |
| 40S ribosomal protein S27 | 9.48 | 2.0×10^7^ | 6 |

**Supplementary Table 6: LC-MS/MS analysis of RTN3-interacting proteins.** Following Co-IP assays of NRVCs using RTN3 antibody, the protein sample were loaded onto SDS-PAGE and then assessed by LC-MS/MS.

|  | Normal weight  n=20 | Obesity  n=20 | P value |
| --- | --- | --- | --- |
|  |  |  |  |
| **Demographics** |  |  |  |
| Age (years) | 62.95±2.03 | 58.20±2.38 | 0.14 |
| Gender（male%） | 60.00 | 60.00 | ＞0.99 |
| BMI | 22.75±0.26 | 32.64±0.36 | ＜0.01 |
| Systolic blood pressure(mmHg) | 123.30±1.63 | 133.70±1.98 | ＜0.01 |
| Diastolic blood pressure(mmHg) | 74.00±0.97 | 79.95±2.58 | 0.04 |
| **Cardiac Function** |  |  |  |
| LAD (mm) | 32.85±0.96 | 39.30±0.78 | ＜0.01 |
| LVEDD (mm) | 46.10±0.83 | 51.65±1.03 | ＜0.01 |
| LVESD (mm) | 30.10±0.70 | 36.40±2.15 | ＜0.01 |
| EF (%) | 65.75±0.01 | 61.55±0.02 | 0.02 |
| E (m/s) | 82.05±2.99 | 66.95±2.60 | ＜0.01 |
| A (m/s) | 79.15±1.90 | 81.20±2.00 | 0.46 |
| E/A | 1.05±0.05 | 0.84±0.04 | ＜0.01 |
| FS (%) | 35.75±0.01 | 33.60±0。01 | 0.01 |
| Cardiac output | 4.20±0.12 | 5.25±0.21 | ＜0.01 |
| **Laboratory values** |  |  |  |
| FPG (mmol/L) | 5.46±0.08 | 6.04±0.11 | ＜0.01 |
| Triglycerides (mmol/L) | 1.28±0.09 | 1.77±0.09 | ＜0.01 |
| Total cholesterol (mmol/L) | 3.69±0.16 | 4.14±0.10 | 0.02 |
| HDL-C (mmol/L) | 1.07±0.03 | 0.99±0.02 | 0.07 |
| LDL-C (mmol/L) | 1.81±0.15 | 2.19±0.08 | 0.03 |
| **Medications** |  |  |  |
| Statins (%) | 71.43 | 85.37 | 0.16 |
| ACE-i/ARBs (%) | 0.00 | 56.10 | ＜0.01 |
| Beta-blockers (%) | 25.00 | 48.78 | 0.04 |
| Diuretics (%) | 17.86 | 43.90 | 0.02 |

**Supplementary Table 7: Demographics, echocardiographic data, laboratory values, and medications in people with normal BMI (healthy control) and obese patients (obesity).** All data were expressed as mean±SEM. P values were calculated using Student t or Chi2 tests. BMI, body mass index; LAD, left atrial diameter; LVEDD, left ventricular end-diastolic diameter; LVESD, left ventricular end-systolic diameter; EF, ejection fraction; FS, fractional shortening; FPG, fasting plasma glucose; LDL-C, low density lipoprotein cholesterol; HDL-C, high density lipoprotein cholesterol; ACE-i, angiotensin converting enzyme inhibitors, ARBs, angiotensin receptor blockers.

**Legends for supplementary video files**

**Supplementary Video 1: Fluorescence image series showing LD in cardiomyocytes infected with Ad NC.** NRVCs were infected with Ad NC and LDs were labeled with Bodipy 493/503. Then NRVCs were then treated with 500μM palmitate for 12 hours. During the palmitate treatment, serial fluorescence images were taken every 1 hour using a confocal laser-scanning microscope. Scale bar = 10μm.

**Supplementary Video 2: Fluorescence image series showing LD in cardiomyocytes infected with Ad RTN3.** NRVCs were infected with Ad RTN3 and LDs were labeled with Bodipy 493/503. Then NRVCs were then treated with 500μM palmitate for 12 hours. During the palmitate treatment, serial fluorescence images were taken every 1 hour using a confocal laser-scanning microscope. Scale bar = 10μm.

**Supplementary Video 3: Fluorescence image series showing LD in cardiomyocytes infected with Ad sh-RTN3.** NRVCs were infected with Ad sh-RTN3 and LDs were labeled with Bodipy 493/503. Then NRVCs were then treated with 500μM palmitate for 12 hours. During the palmitate treatment, serial fluorescence images were taken every 1 hour using a confocal laser-scanning microscope. Scale bar = 10μm.
