# Supplementary figures and images for: Lipid overload-induced RTN3 activation leads to cardiac dysfunction by promoting lipid droplet biogenesis
Source: Cell Death Differ. 2023 Nov 28;31(3):292–308. doi: 10.1038/s41418-023-01241-x (PMC10923887; doi:10.1038/s41418-023-01241-x)

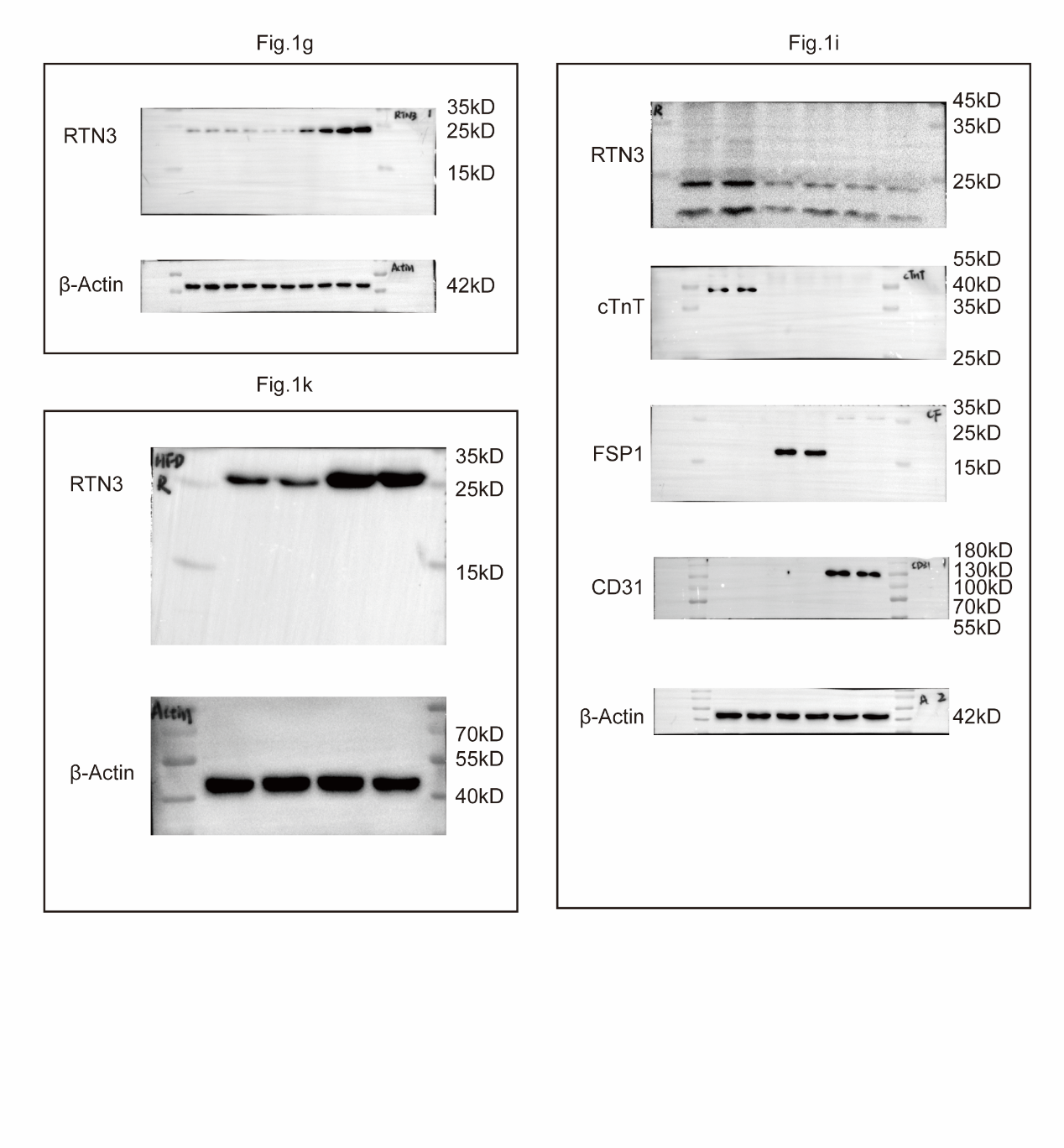


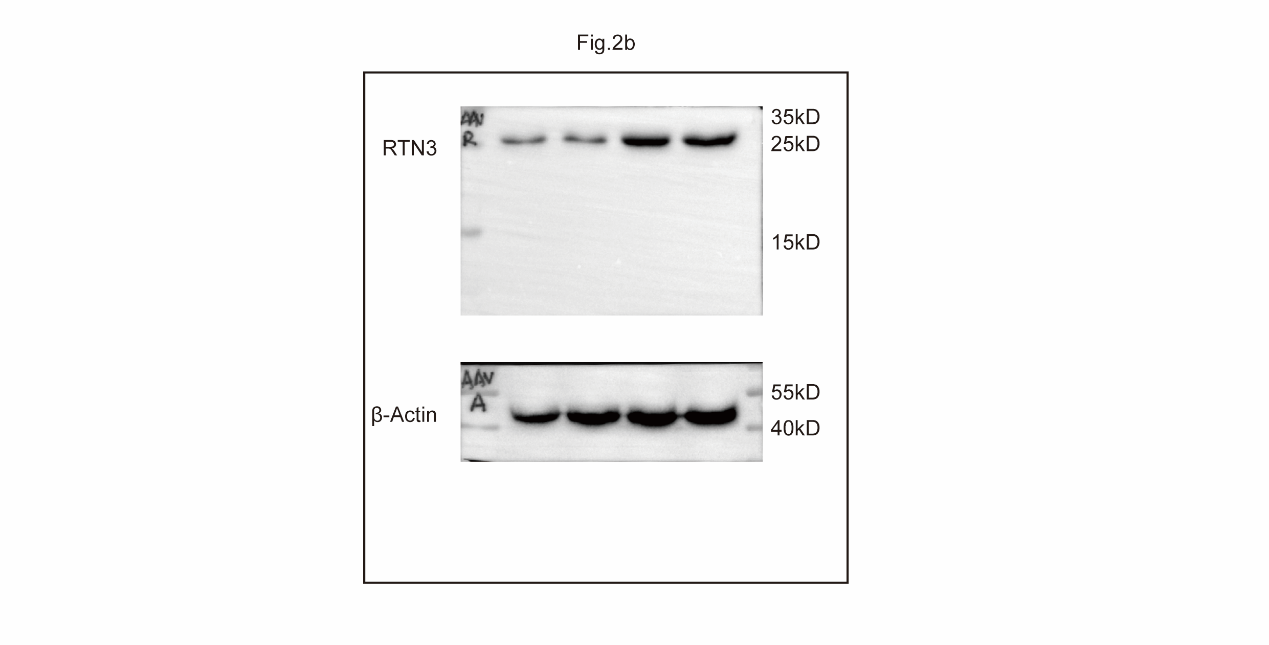


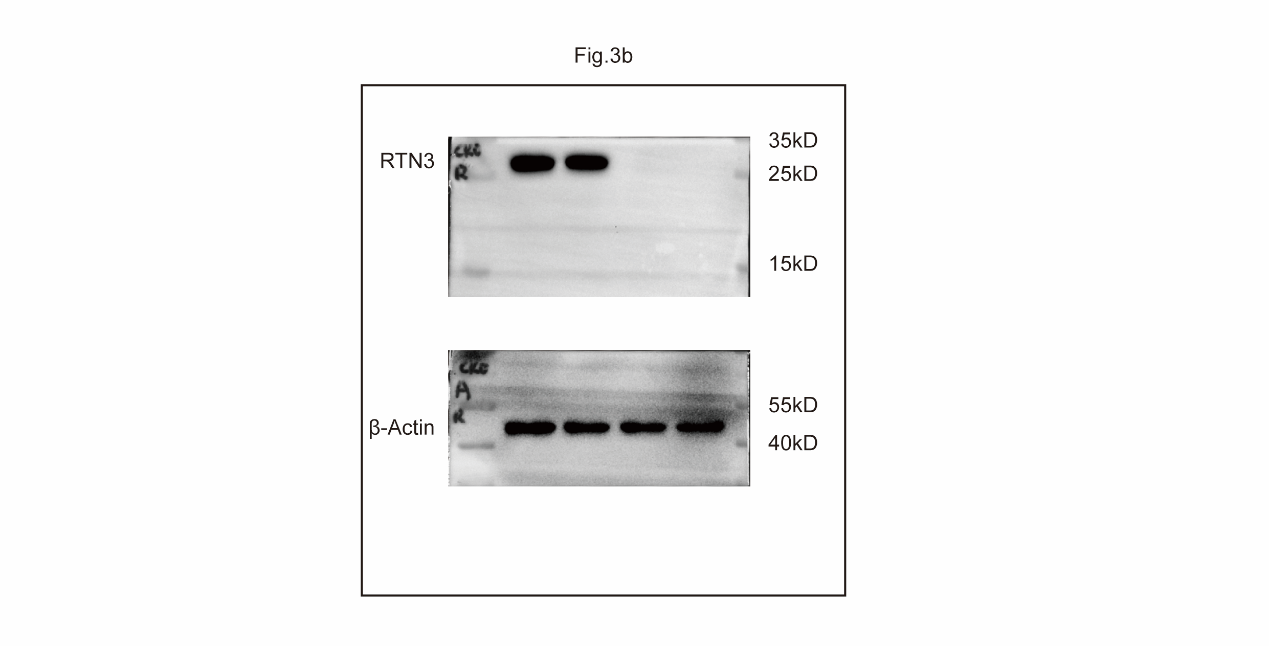


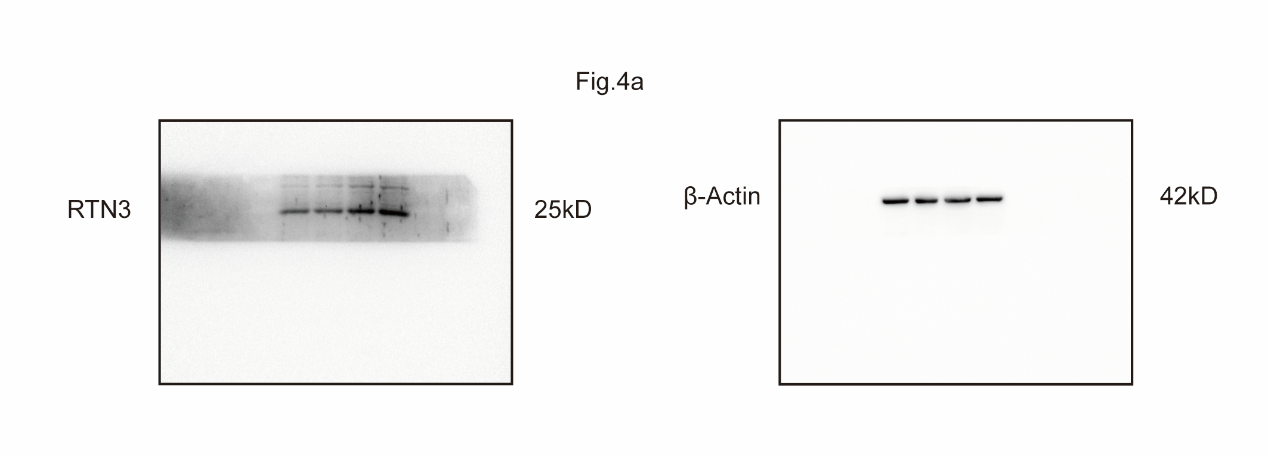


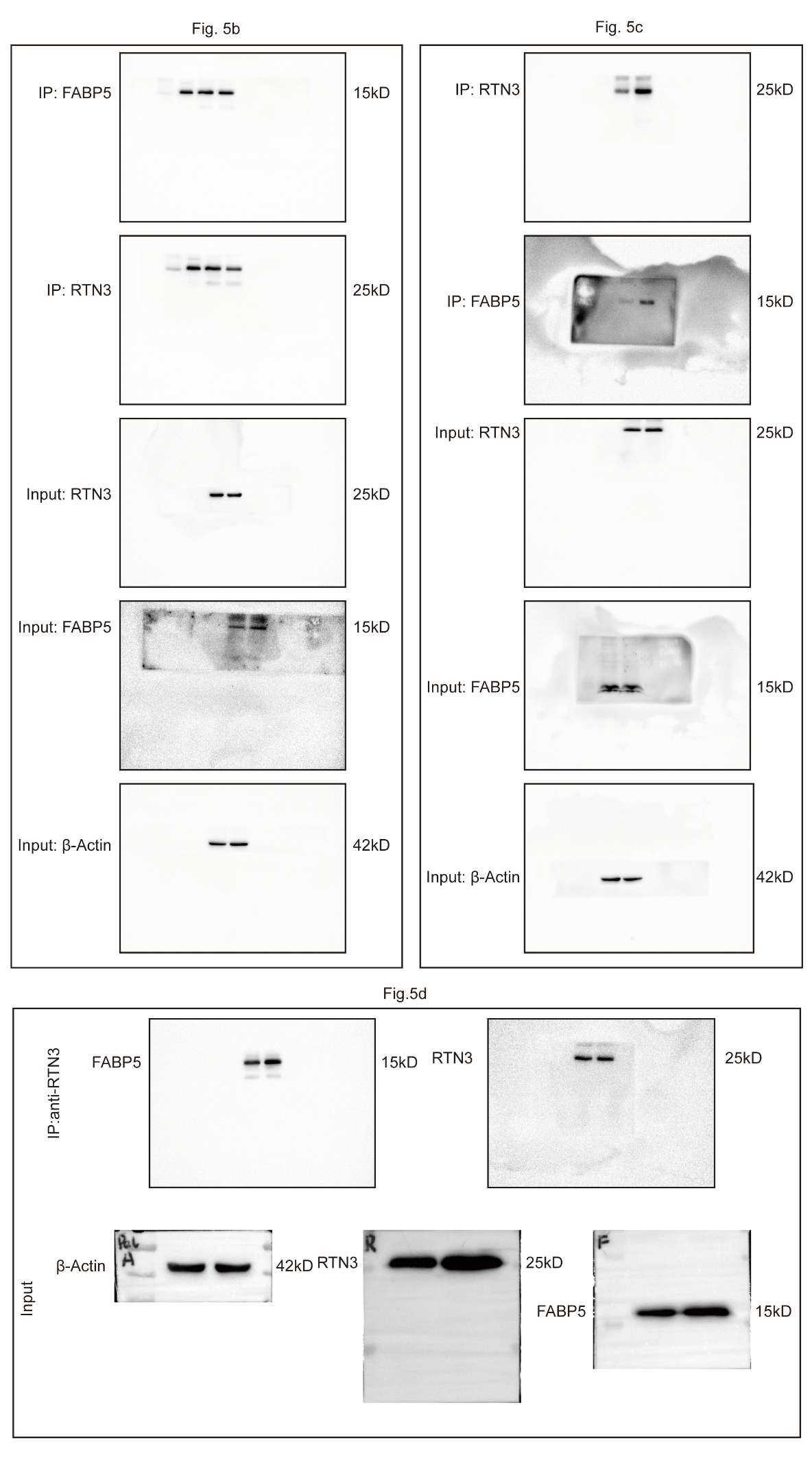


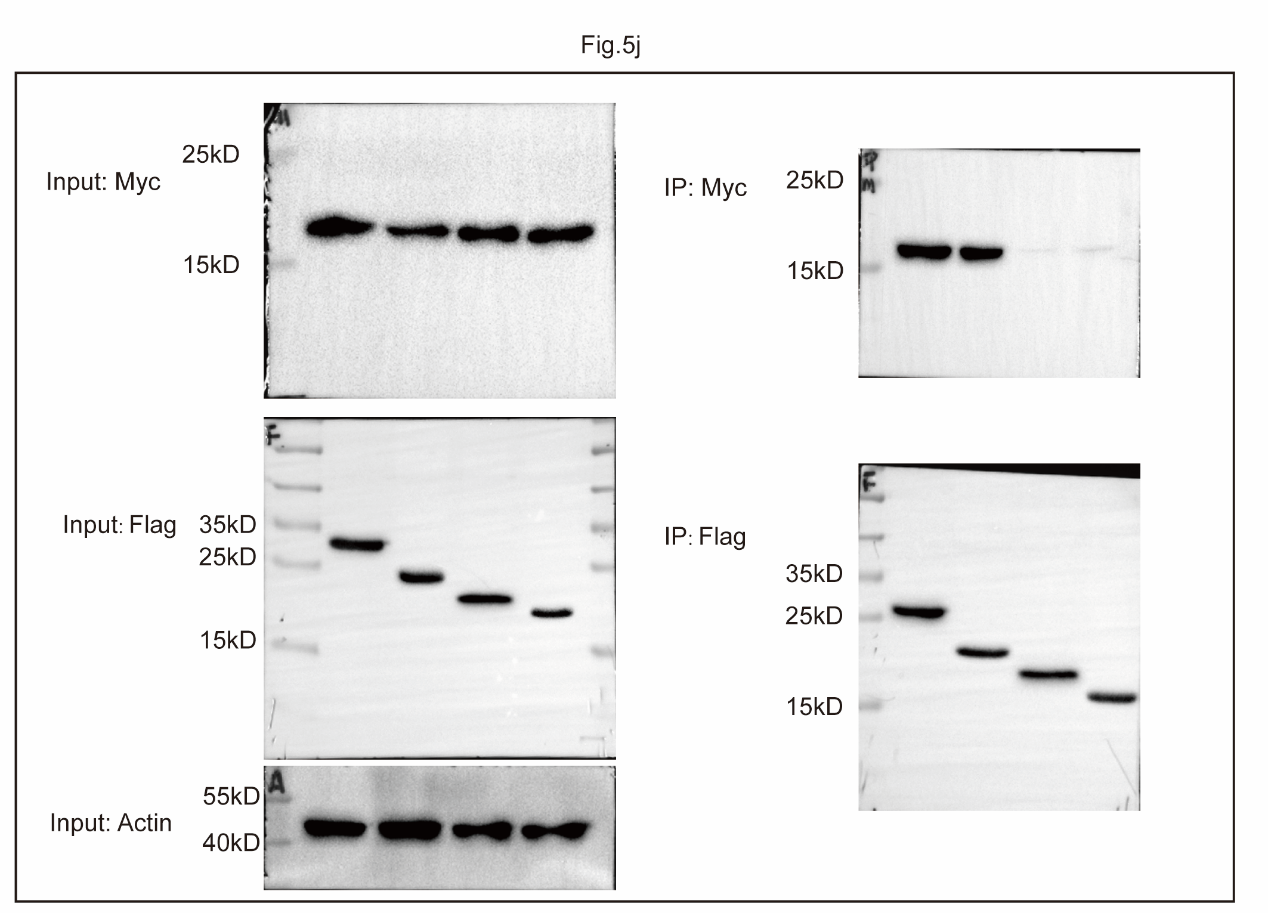


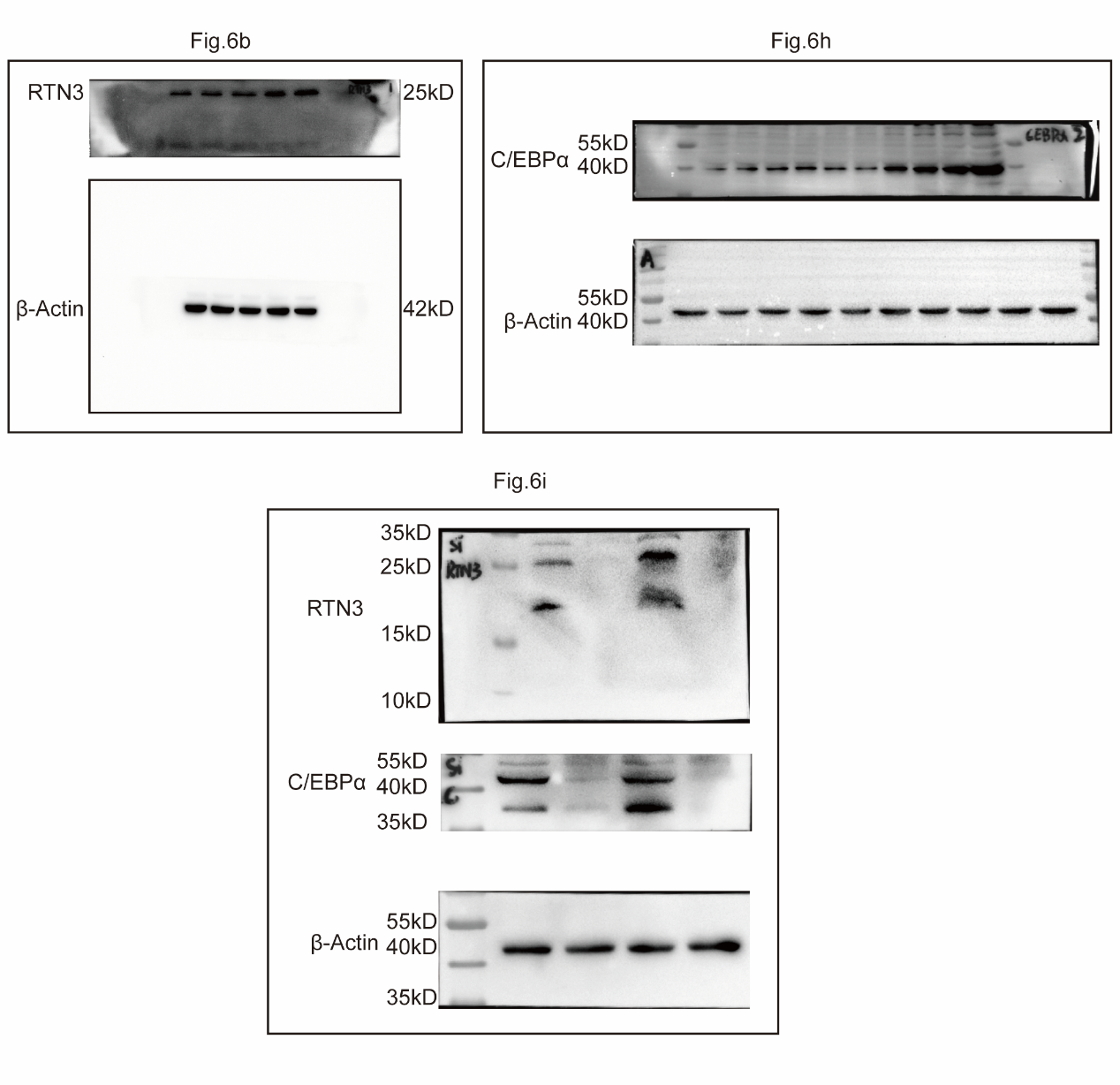


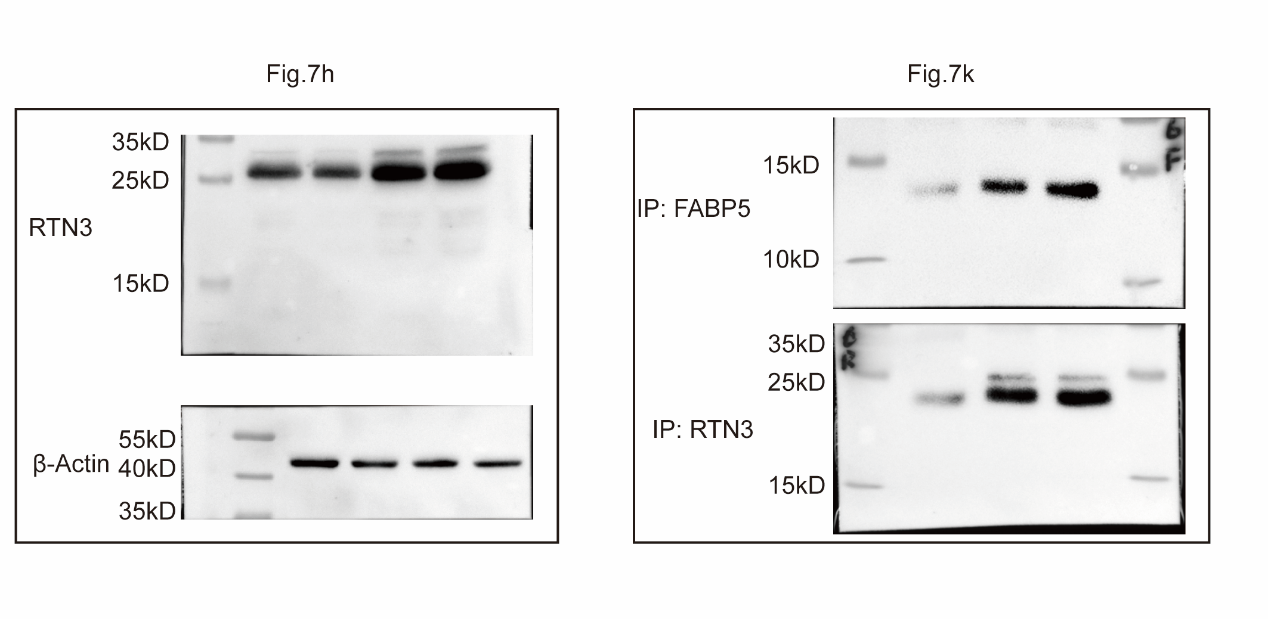


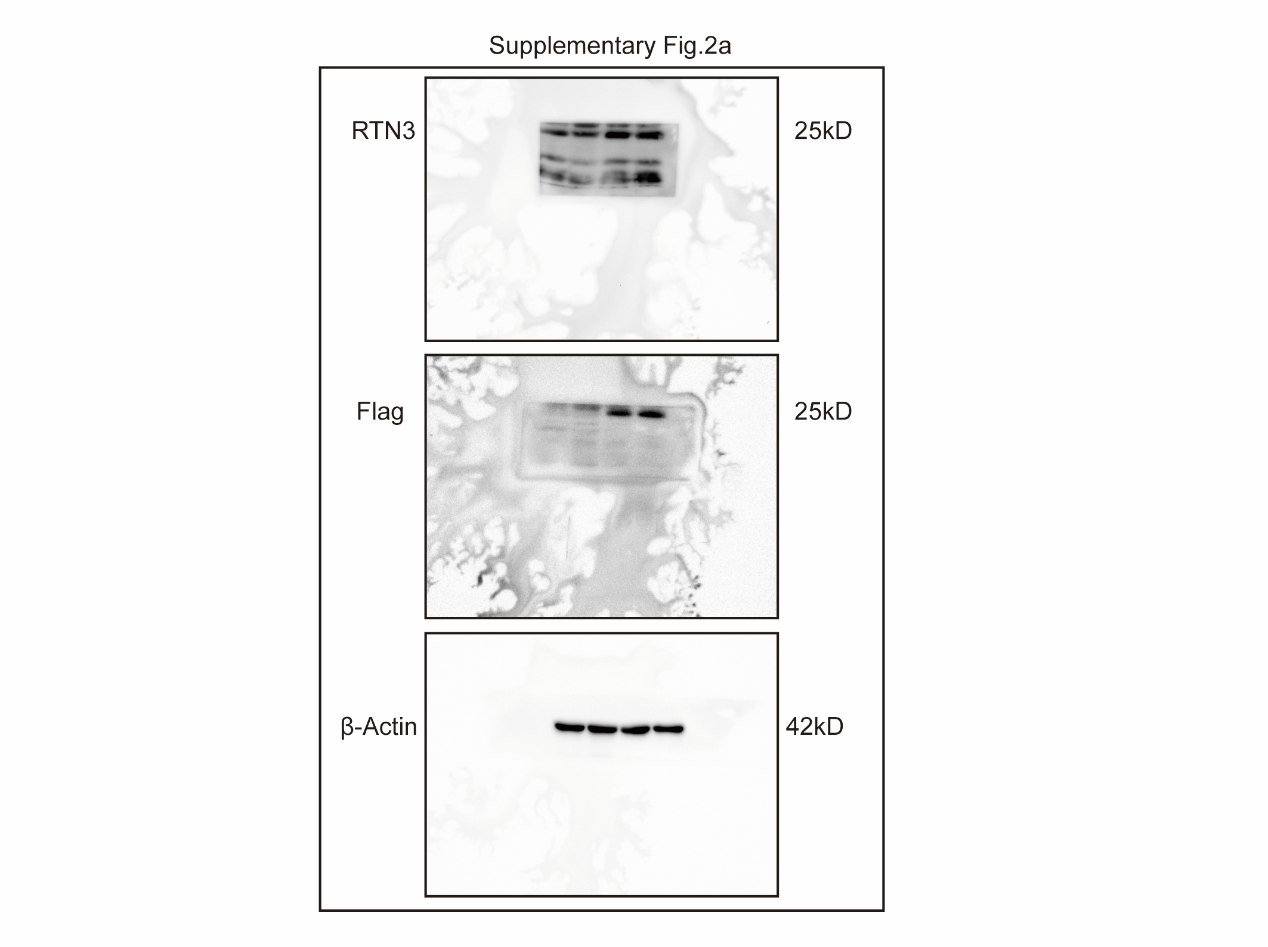


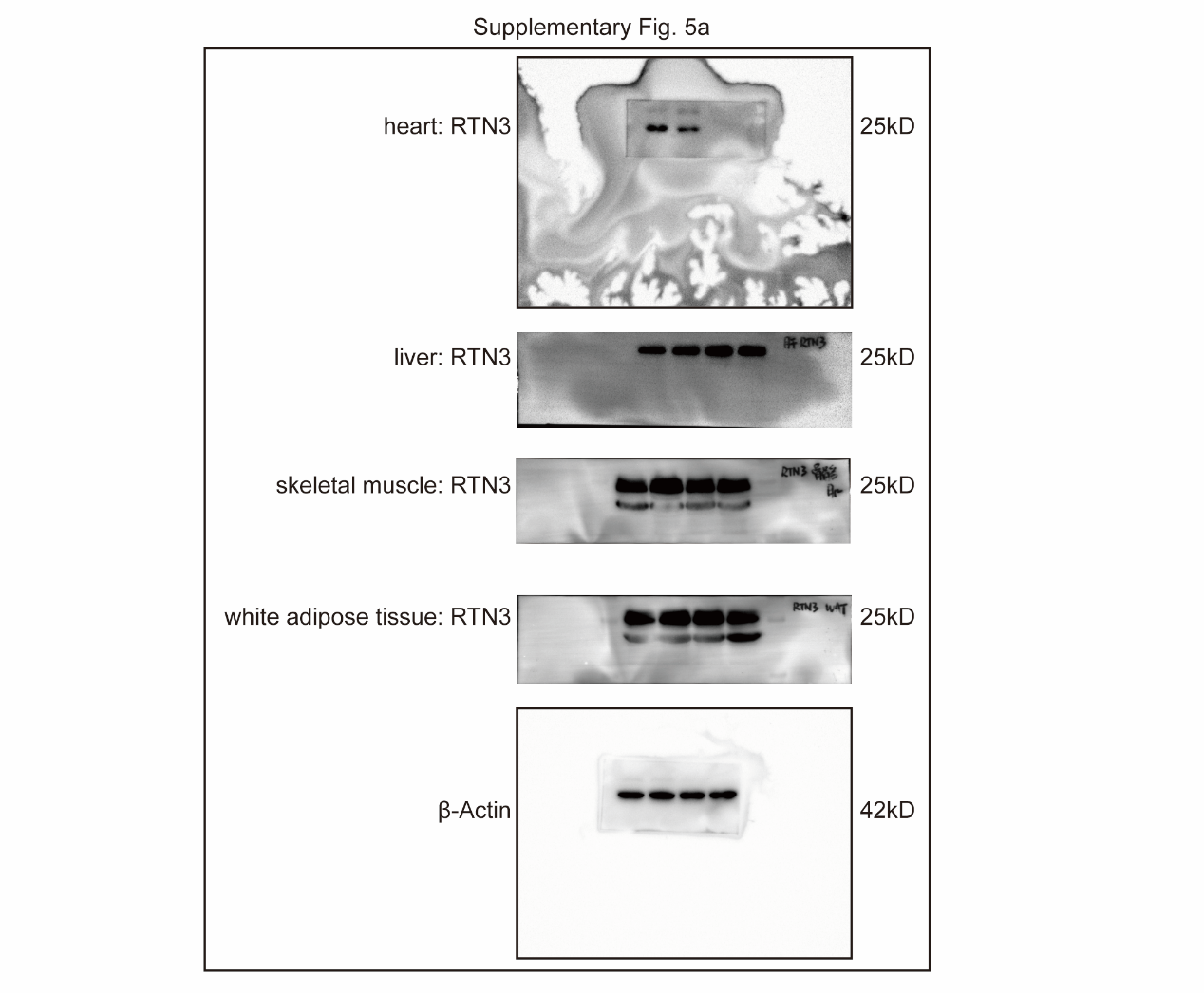


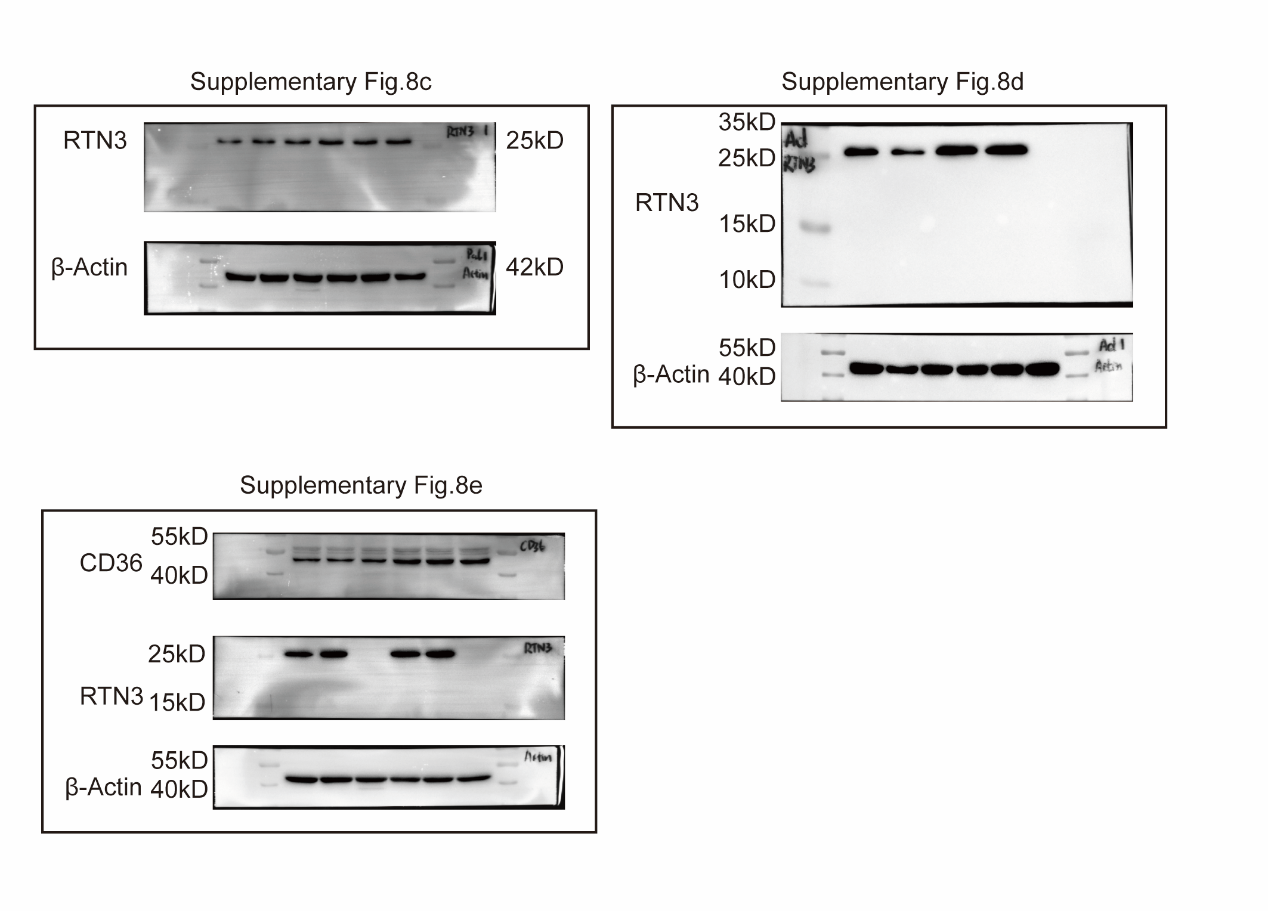


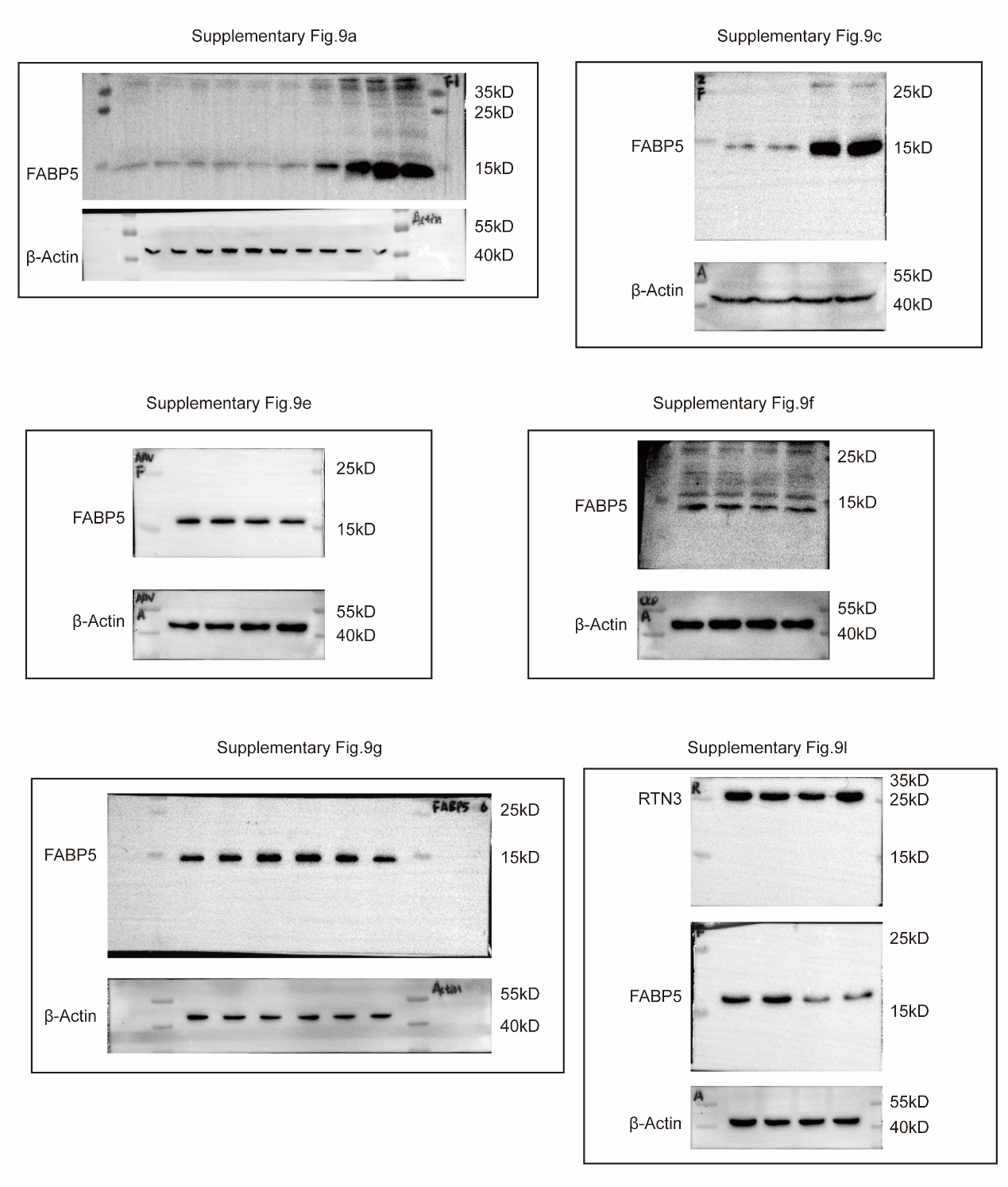


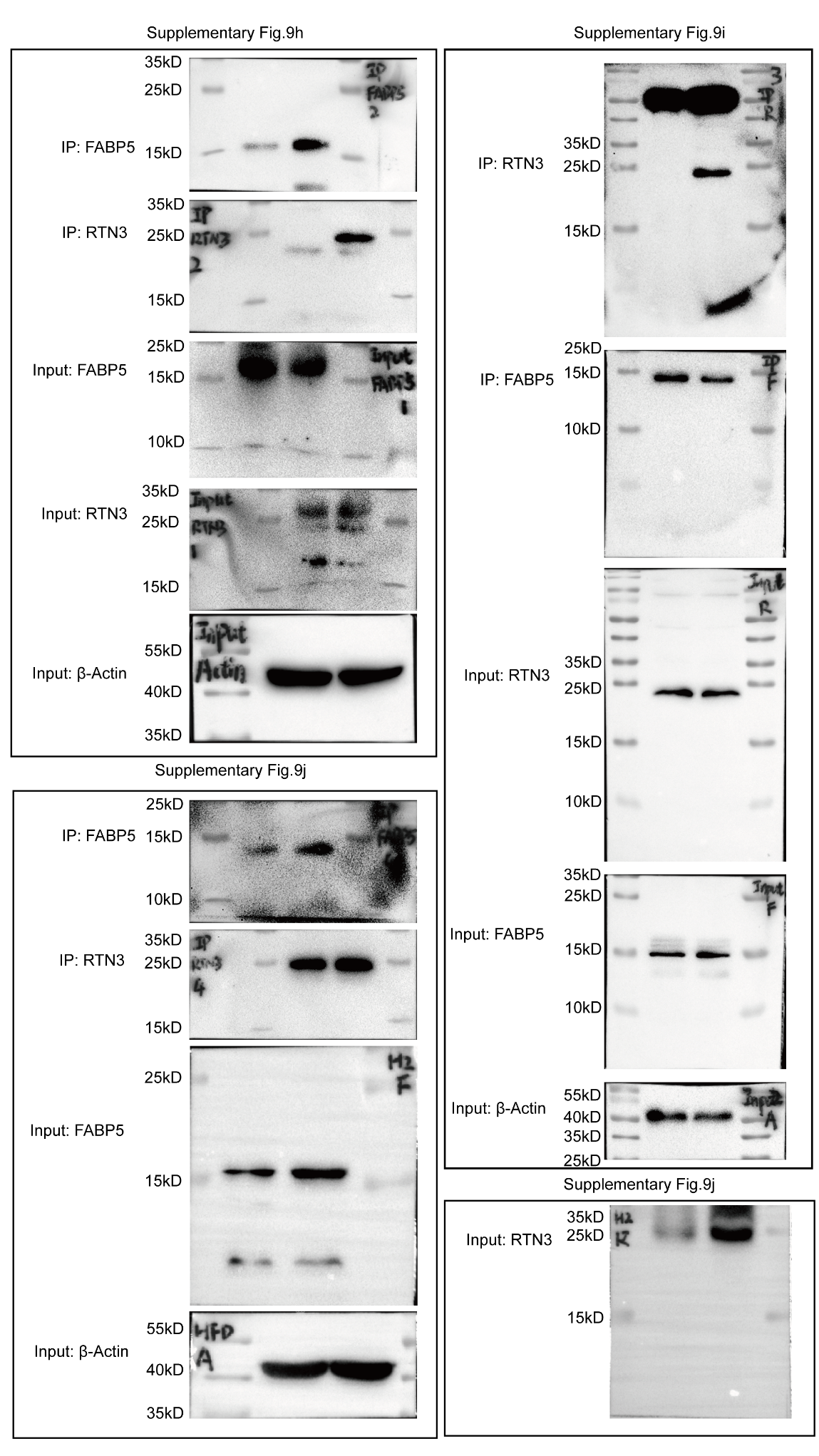


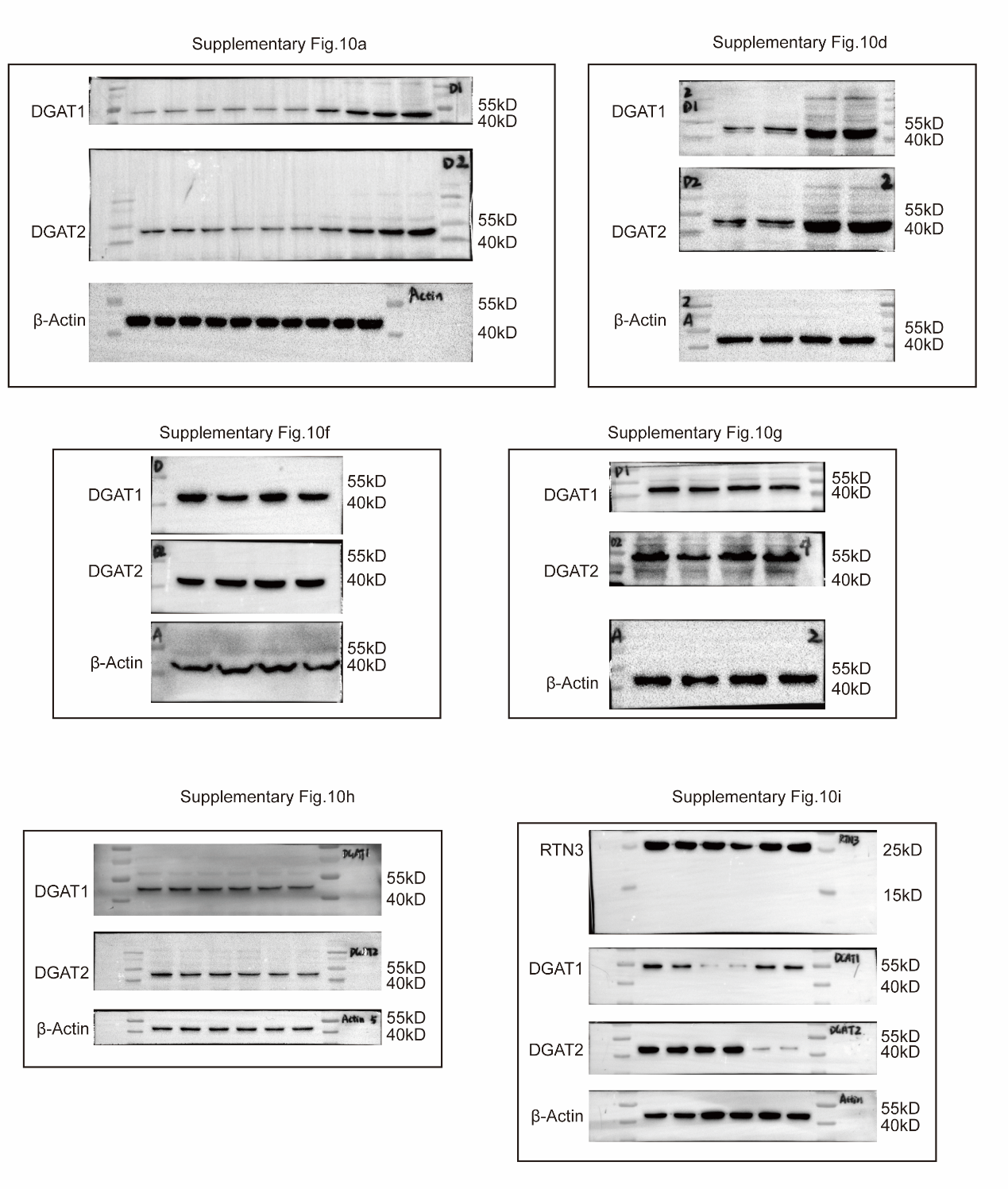

Supplement: Supplementary file 5 — Original Data File [file 41418_2023_1241_MOESM5_ESM.docx]
